# Biosensor-driven, model-based optimization of the orthogonally expressed naringenin biosynthesis pathway

**DOI:** 10.1186/s12934-022-01775-8

**Published:** 2022-03-27

**Authors:** Maarten Van Brempt, Andries Ivo Peeters, Dries Duchi, Lien De Wannemaeker, Jo Maertens, Brecht De Paepe, Marjan De Mey

**Affiliations:** Centre For Synthetic Biology, Ghent University, Coupure Links 653, B-9000 Ghent, Belgium

**Keywords:** Metabolic engineering, Orthogonality, Statistical learning, Escherichia coli, Flavonoid, Transcriptional biosensor

## Abstract

**Background:**

The rapidly expanding synthetic biology toolbox allows engineers to develop smarter strategies to tackle the optimization of complex biosynthetic pathways. In such a strategy, multi-gene pathways are subdivided in several modules which are each dynamically controlled to fine-tune their expression in response to a changing cellular environment. To fine-tune separate modules without interference between modules or from the host regulatory machinery, a sigma factor (σ) toolbox was developed in previous work for tunable orthogonal gene expression. Here, this toolbox is implemented in *E. coli* to orthogonally express and fine-tune a pathway for the heterologous biosynthesis of the industrially relevant plant metabolite, naringenin. To optimize the production of this pathway, a practical workflow is still imperative to balance all steps of the pathway. This is tackled here by the biosensor-driven screening, subsequent genotyping of combinatorially engineered libraries and finally the training of three different computer models to predict the optimal pathway configuration.

**Results:**

The efficiency and knowledge gained through this workflow is demonstrated here by improving the naringenin production titer by 32% with respect to a random pathway library screen. Our best strain was cultured in a batch bioreactor experiment and was able to produce 286 mg/L naringenin from glycerol in approximately 26 h. This is the highest reported naringenin production titer in *E. coli* without the supplementation of pathway precursors to the medium or any precursor pathway engineering. In addition, valuable pathway configuration preferences were identified in the statistical learning process, such as specific enzyme variant preferences and significant correlations between promoter strength at specific steps in the pathway and titer.

**Conclusions:**

An efficient strategy, powered by orthogonal expression, was applied to successfully optimize a biosynthetic pathway for microbial production of flavonoids in *E. coli* up to high, competitive levels. Within this strategy, statistical learning techniques were combined with combinatorial pathway optimization techniques and an in vivo high-throughput screening method to efficiently determine the optimal operon configuration of the pathway. This “pathway architecture designer” workflow can be applied for the fast and efficient development of new microbial cell factories for different types of molecules of interest while also providing additional insights into the underlying pathway characteristics.

**Supplementary Information:**

The online version contains supplementary material available at 10.1186/s12934-022-01775-8.

## Background

Following the advances in metabolic engineering and synthetic biology, an increasing interest emerged over the past decades in microbial production as a valuable alternative for conventional production methods of numerous and diverse (bio) chemicals [1–7]. Efforts have focused on tackling the bottlenecks in the biosynthesis of both native and heterologous products to unlock its industrial potential. Synthetic biology especially, unlocks the potential to deal with the optimization of complex heterologous pathways through the implementation of a synthetic regulatory layer, (dynamically) controlling the flux through different modules of the pathway. For this purpose, in previous work, a regulatory “sigma (σ) factor toolbox” was created which enables tunable expression of up to three different modules, in an orthogonal (i.e. independent of each other, without crosstalk) manner [8]. Orthogonal pathway construction holds significant advantages over their non-orthogonal counterparts for the creation of microbial cell factories producing (heterologous) compounds of interest [9–12].

One such a class of complex (bio) chemicals sparking industrial interest is flavonoids, which are naturally produced in plants by an elaborate network of biosynthetic pathways. To date, over 9000 of these specialized plant metabolites have been identified, which display a wide variety of biological activities with industrial application [13, 14]. Centrally positioned in the flavonoid biosynthesis network is the metabolite naringenin, which is used as a scaffold molecule for further enzymatic processing with numerous chemical decorations and modifications [15]. Its relevance already led to many engineering efforts to create an efficient naringenin producing microbial cell factory (MCF), either by focusing on improving precursor molecule supply by deleting or knocking down genes of enzymes consuming these molecules, overexpression of genes producing them, the construction of the heterologous pathway using different isozymes and expression levels, or a combination of strategies [5, 16–25]. These factors make naringenin an interesting initial target for the construction of an orthogonally expressed pathway module, to which additional modules can be connected, expanding the genetic network for the custom biosynthesis of various flavonoids.

The optimization of the heterologous biosynthesis of flavonoids is in no way straightforward and despite these previous engineering efforts, its industrial potential still remains largely unexploited [3, 16]. To engineer MCFs for maximal productivitiy it is required to attune every enzymatic reaction in the pathway to each other and to the cell’s available resources, thereby avoiding flux imbalances leading to accumulation of metabolites and associated toxicity or a potential detrimental metabolic burden on the cell [26–28]. At present, the advancements in DNA synthesis and numerous existing DNA assembly techniques support the possibility of a combinatorial engineering approach, which integrates a whole set of varying genetic parts in a single assembly, resulting in large libraries of pathway variants [29]. Though this approach enables the creation of strains with higher productivities, the size of the search space grows exponentially with the number of used parts and length of the pathway:

1 $$\# pathway\,variants = \left( {\# promoters*\# RBSs*\# enzyme\,variants*\# terminators} \right)^{\# operons\,\,in\,the\,pathway}$$

(assuming monocistronic operons), rendering it very challenging to find the “metabolic sweet spot”. Therefore, in the past decade, statistical, and more recently, machine learning (ML) methods gradually found their way in synthetic biology with respect to biosynthetic pathway optimization [30, 31]. These methods include, i.a., procedures to improve the experimental design, limiting the required practical throughput of experiments and the creation of stochastic models to allow predictions of optimized pathway architecture for rational engineering. Rather than endlessly screening for the desired strain phenotype in the vast genotypic search space, a more efficient approach is to acquire a small characterized subset of different pathway architectures with corresponding production titers from which the key determinants for pathway performance can be deduced. Computer models aid in identifying complex interactions between pathway features and their correlation with product synthesis to ultimately predict the potential of new compositions with the used genetic building blocks. This learning process can be repeated in multiple Design-Build-Test-Learn (DBTL) cycles [32] by testing the top predictions and adding these as input to the next cycle, rapidly converging toward the optimal pathway architecture, and thereby decreasing the experimental load.

As shown by Zhou et al. [30], the quality of the (initial) data being fed to a model is crucial for the accuracy of the predicted pathway performance and thus the overall success of the engineering strategy. To collect high-quality data, rather than characterizing a small randomly selected library, a pre-screen is required to select a variety of producing phenotypes. This implies the need for a high-throughput screen to avoid a laborious and time-consuming selection process. For many molecules, as is the case for naringenin, no obvious screen, e.g. colorimetric measurement, is available. In this respect, small molecule-responsive transcriptional biosensors are a very valuable tool [33].

**Fig. 1:**
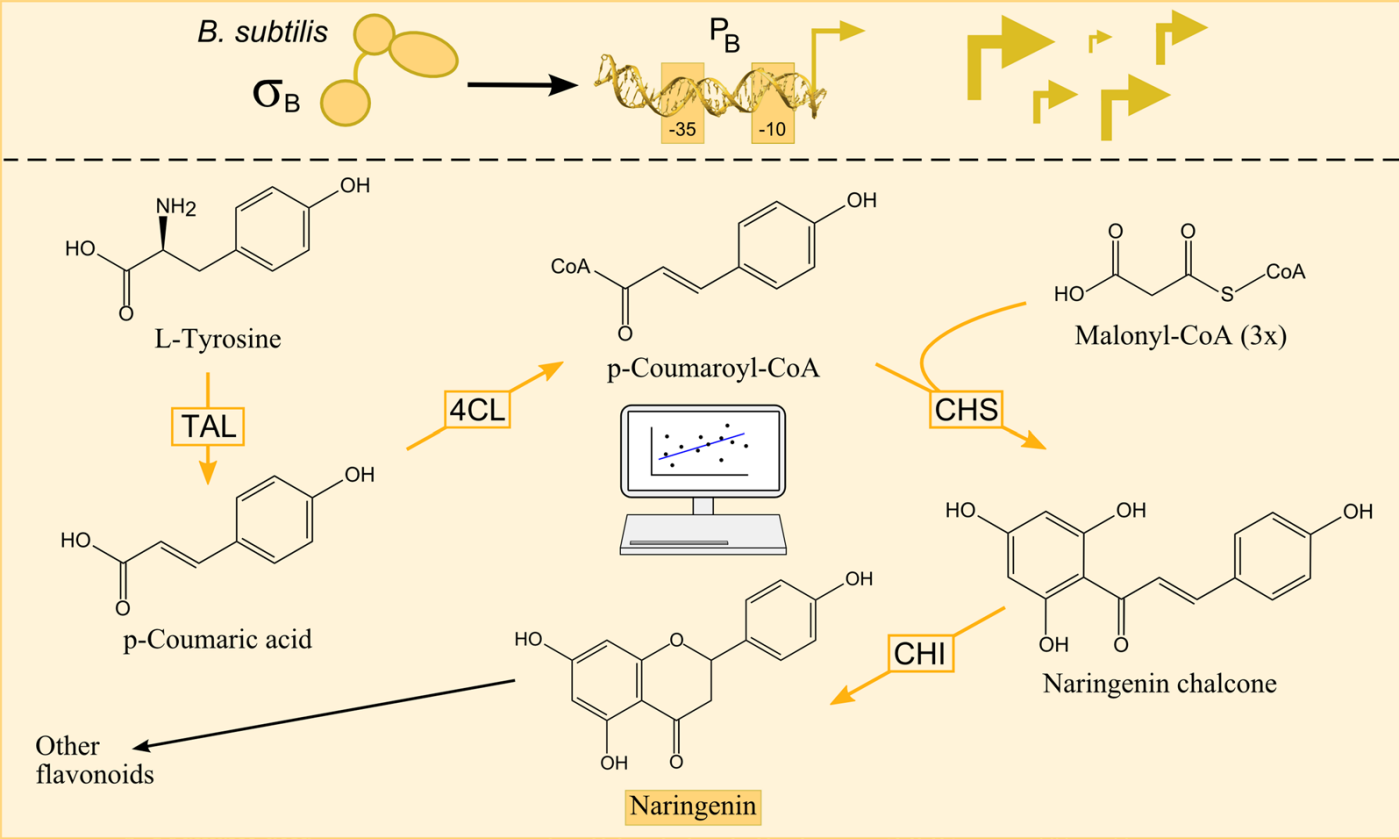
Overview of the orthogonally expressed naringenin production module. The pathway is driven by σ^B^-specific promoters and production is optimized with a combinatorial engineering approach followed by predictive modeling. (*TAL* Tyrosine ammonia-lyase, *4CL* 4-coumaroyl-CoA ligase, *CHS* Chalcone synthase, *CHI* Chalcone isomerase, *B. subtilis*: *Bacillus subtilis*, *σ* sigma factor, *P*_*B*_ σ^B^-specific promoter from the library of Bervoets et al*.* 2018 [8], originating from *B. subtilis*)

In this work, a combinatorial engineering approach using the tools for tunable and orthogonal expression, created in Bervoets et al. (2018) [8], is combined with a biosensor-driven screening to collect high-quality data to feed three different predictive models with increasing complexity to optimize microbial naringenin biosynthesis (see Fig. 1). As multiple mathematical tools offer a solution to metabolic optimization questions, the added value of more complex models is assessed with the potential trade-offs such as overfitting risks or output information content. Subsequently, the performance of the best candidate strain is validated on bioreactor scale.

## Results

### Combinatorial pathway assembly, screening and data collection

To create a naringenin producing module in *Escherichia coli* (*E. coli*), four non-native catalytic reactions are required, starting from two precursor molecules which are naturally present in *E. coli*, i.e., L-tyrosine and malonyl-CoA (see Fig. 1). These four reactions are mediated by a tyrosine ammonia-lyase (TAL), a 4-coumaroyl-CoA ligase (4CL) a chalcone synthase (CHS) and a chalcone isomerase (CHI).

**Fig. 2:**
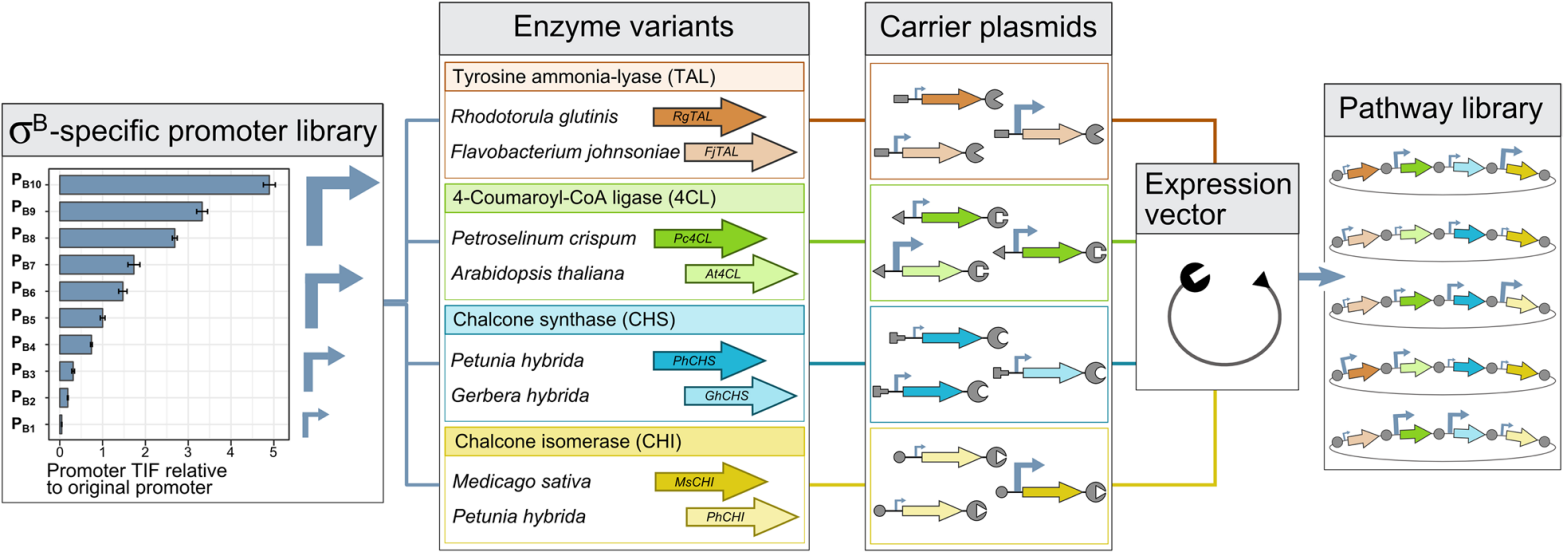
Schematic representation of the combinatorial construction of the naringenin biosynthesis pathway. For every catalytic step in the pathway, libraries are created on carrier plasmids, each library variant being composed of one out of ten different σ^B^-specific promoters and one out of two different enzyme variants. All carrier plasmid inserts are subsequently assembled in a predefined order in a single reaction, generating a pathway library [34]. (*TIF* transcription initiation frequeny, *σ* sigma factor)

The described module is assembled in a combinatorial manner, with variability introduced at the promoter and enzymatic levels. The σ^B^-specific promoter library from Bervoets et al. (2018) [8] drives orthogonal gene transcription and comprises 10 different promoter variants with variable transcription initiation frequency (TIF) (see Fig. 2). At the enzymatic level, two isozymes were selected for each step of the pathway based on their reported ability to catalyze the specific reactions in *E. coli* (see Fig. 2 and Table 2). As such, the theoretical search space comprises 160.000 (see Eq. 1) possible pathway configurations. For the construction, a Golden Gate (GG) based assembly procedure is performed as described by Coussement et al*.* (2017) [34]. First, for each enzyme type, a carrier plasmid library is created (four in total), containing random promoter–isozyme combinations, with each operon surrounded by carrier plasmid-specific linkers. These linkers include sequentially matching GG-sites to allow the merger of the operon libraries in an expression vector to be in a predefined order, and with each enzyme type only occurring once in a single pathway variant (see Fig. 2). For the construction of the carrier plasmid libraries, cross-lapping in vitro assembly (CLIVA) was used [35]. This method, similar to GG, is sequence-independent and together with the σ^B^-dependency of promoter expression, it ensures that the cloning aspect of the combinatorial engineering approach does not favor integration of specific parts over another and that no bias is created as a result of growth speed differences caused by overexpression-related metabolic burden. The occurrence of specific σ^B^ promoter–isozyme combinations in the following selection process should therefore solely be a result of the naringenin production capacity and not be tied to the library preparation.

**Fig. 3:**
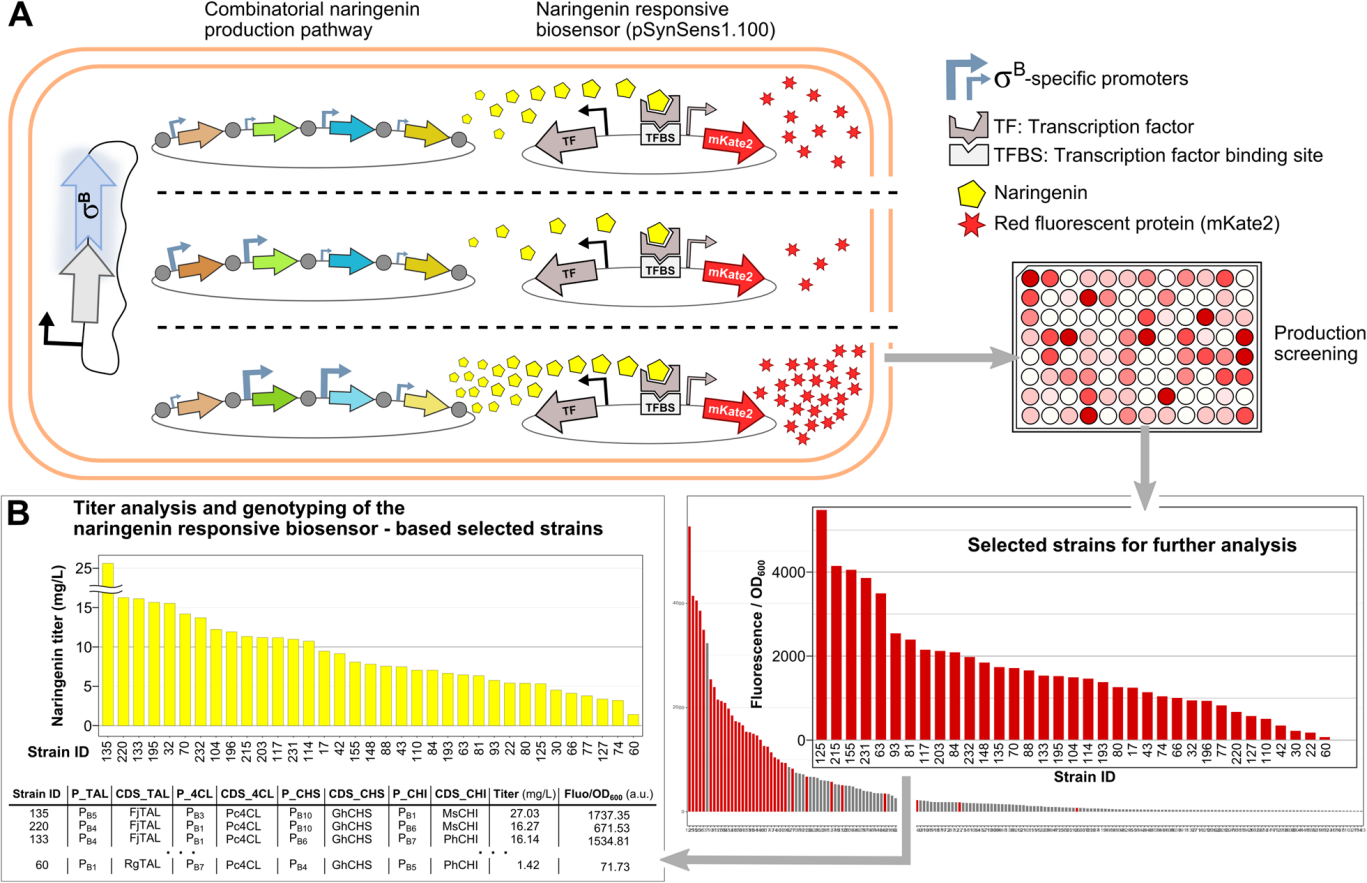
Overview of the naringenin production screening, strain selection and characterization process. **A** A strain harboring the heterologous sigma factor B (σ^B^) from *Bacillus subtilis* in the genome (inserted in the rpoS operon) [8], was cotransformed with the naringenin biosynthetic pathway library and the naringenin-responsive biosensor plasmid (pSynSens1.100) [36]. The strains are screened for naringenin production on microtiter plate-scale through their biosensor-generated fluorescence. **B** 35 strains were selected that are predicted to cover a wide range of naringenin production titers. These strains were further characterized individually using UPLC analysis to measure the actual naringenin product titers and sequence analysis was used to reveal the incorporated promoters (P_B1_ to P_B10_) and coding sequences (CDS) of the isozymes. The characteristics of all 35 strains together with the measured fluorescence/OD_600_ are given in Additional file 1: Table S1. (Rg: *Rhodotorula glutinis*; Fj: *Flavobacterium johnsoniae*; Pc: *Petroselinum crispum*; At: *Arabidopsis thaliana*; Ph: *Petunia hybrida*; Gh: *Gerbera hybrida*; Ms: *Medicago sativa*; TAL: Tyrosine ammonia-lyase; 4CL: 4-coumaroyl-CoA ligase; CHS: Chalcone synthase; CHI: Chalcone isomerase)

The assembly mix containing the pathway library was then introduced into the *E. coli* strain harbouring the heterologous σ^B^ in the genome [8]. This strain also harbored pSynSens1.100, the naringenin-responsive biosensor plasmid described by De Paepe et al. (2018) [36]. The latter enables the selection of naringenin-producing strains based on an easy-to-measure fluorescent signal, generated by the biosensor in response to the present naringenin concentration (see Fig. 3A). A random selection of 190 colonies was screened on a microtiter plate (MTP)-scale and simultaneously the naringenin-responsive biosensor was characterized. The biosensor’s relationship between naringenin concentration and fluorescence, and its parameterized properties derived from a Hill function fit, are depicted in Additional file 1: Fig. S1. The acquired fluorescence data from the 190 different strains was normalized for optical density (OD_600_) and sorted in descending order. By selecting a subset of strains covering the whole range of produced fluorescence (as a measure for naringenin production) for further characterization, the relevant information content for a fixed subset size is maximized (see Fig. 3B). To obtain a quality dataset to train the mathematical models while maintaining the practical feasibility of further analysis, the selection was restricted to 35 strains.

Of the selected strains, the naringenin titer in the MTP-scale cultures was determined through UPLC analysis and ranged from 1.52 to 27.03 mg/L (see Fig. 3B). Additionally, DNA sequencing was performed to determine the corresponding genotypes. Additional file 1: Table S1 shows for each strain in the subset the specific promoter and enzyme variant combinations, the achieved production titer and the corresponding fluorescent signal generated by the biosensor. For one out of the 35 observations, sequencing was unsuccessful. The employed biosensor-driven combinatorial engineering strategy allowed the selection of a subset of strains, which indeed exhibits a range of production titers resulting from diverse, and each unique, pathway architectures.

### Building computer models as a tool to enchance biosynthesis through a multi-gene pathway

#### Data exploration

To optimize the microbial biosynthesis of naringenin, the acquired data can be used to train a model that predicts the optimal pathway configuration based on the eight input variables: promoter transcription initiation frequency (TIF) of the four enzymatic pathway steps (P_TAL, P_4CL, P_CHS, P_CHI) and the amino acid coding sequence variant of those four enzymes (CDS_TAL, CDS_4CL, CDS_CHS, CDS_CHI). Preceding the model building, the data was explored to expose potential correlation between the predictors and to perform an initial identification of key variables that determine pathway efficiency.

To render the data fit for analysis, the promoter labels (P_B1-10_) [8] were replaced by their TIF values and a linear-logarithmic (linlog) transformation was applied to better describe the magnitude differences in cellular response generated by the different promoters and concordantly reduce model prediction errors significantly [37, 38]. The linlog transformed data is depicted in Additional file 1: Fig. S2.

Correlation between the continuous variables (P_*X* and Titer, with *X* = TAL, 4CL, CHS, CHI) was tested for by determining the Pearson correlation coefficient and statistical testing for significance (see Additional file 1: Fig. S3). Based on a p-value threshold of 0.05, it cannot be stated that there is a linear correlation in the dataset between the P_*X* predictors. Although not significant on a significance level of 0.05 when adjusted for multiple testing (Holm-Bonferroni), we can see indications of a positive correlation between P_TAL and Titer, between P_CHS and Titer and a negative correlation between P_4CL and Titer.

**Fig. 4:**
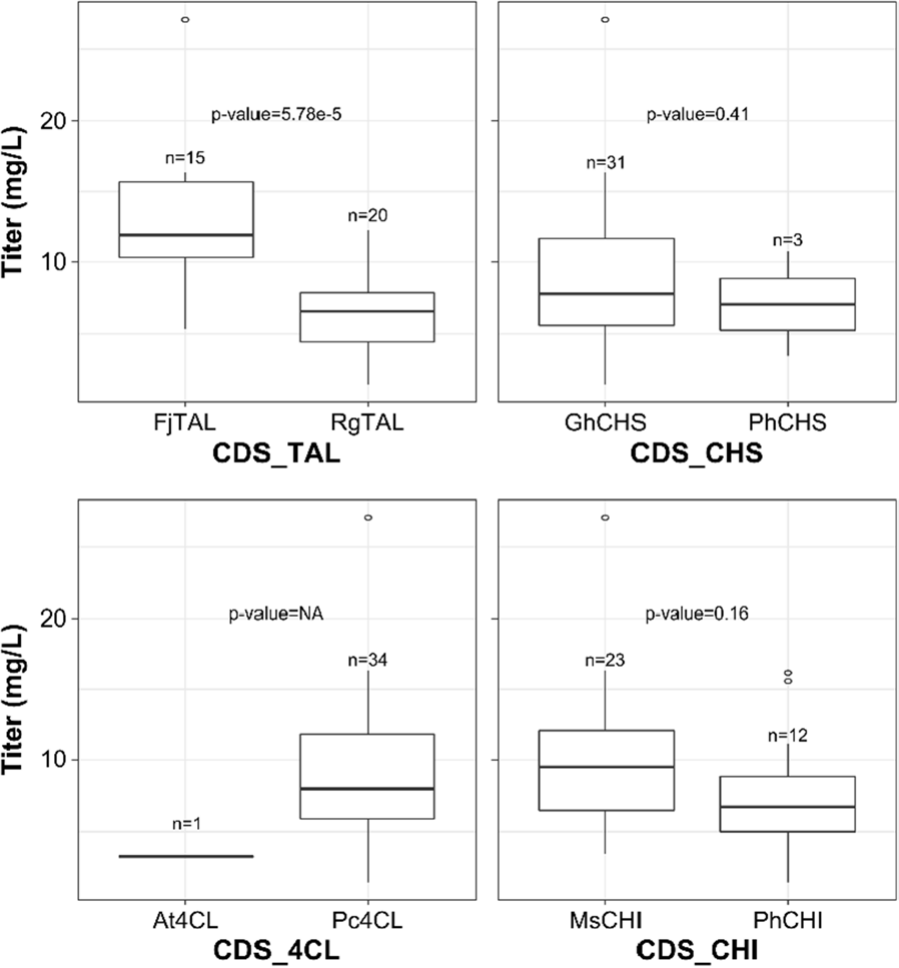
Barplots visualizing the occurrence frequency of either of the isozymes in the characterized pathways versus the achieved naringenin titer with those pathways. p-values are generated by one-way analysis of variance (ANOVA) and indicate a significant difference in the average effect of enzyme variant on naringenin product titer for p < 0.05. (CDS: coding sequence; NA: not applicable; Rg: *Rhodotorula glutinis*; Fj: *Flavobacterium johnsoniae*; Pc: *Petroselinum crispum*; At: *Arabidopsis thaliana*; Ph: *Petunia hybrida*; Gh: *Gerbera hybrida*; Ms: *Medicago sativa*; TAL: Tyrosine ammonia-lyase; 4CL: 4-coumaroyl-CoA ligase; CHS: Chalcone synthase; CHI: Chalcone isomerase)

**Fig. 5:**
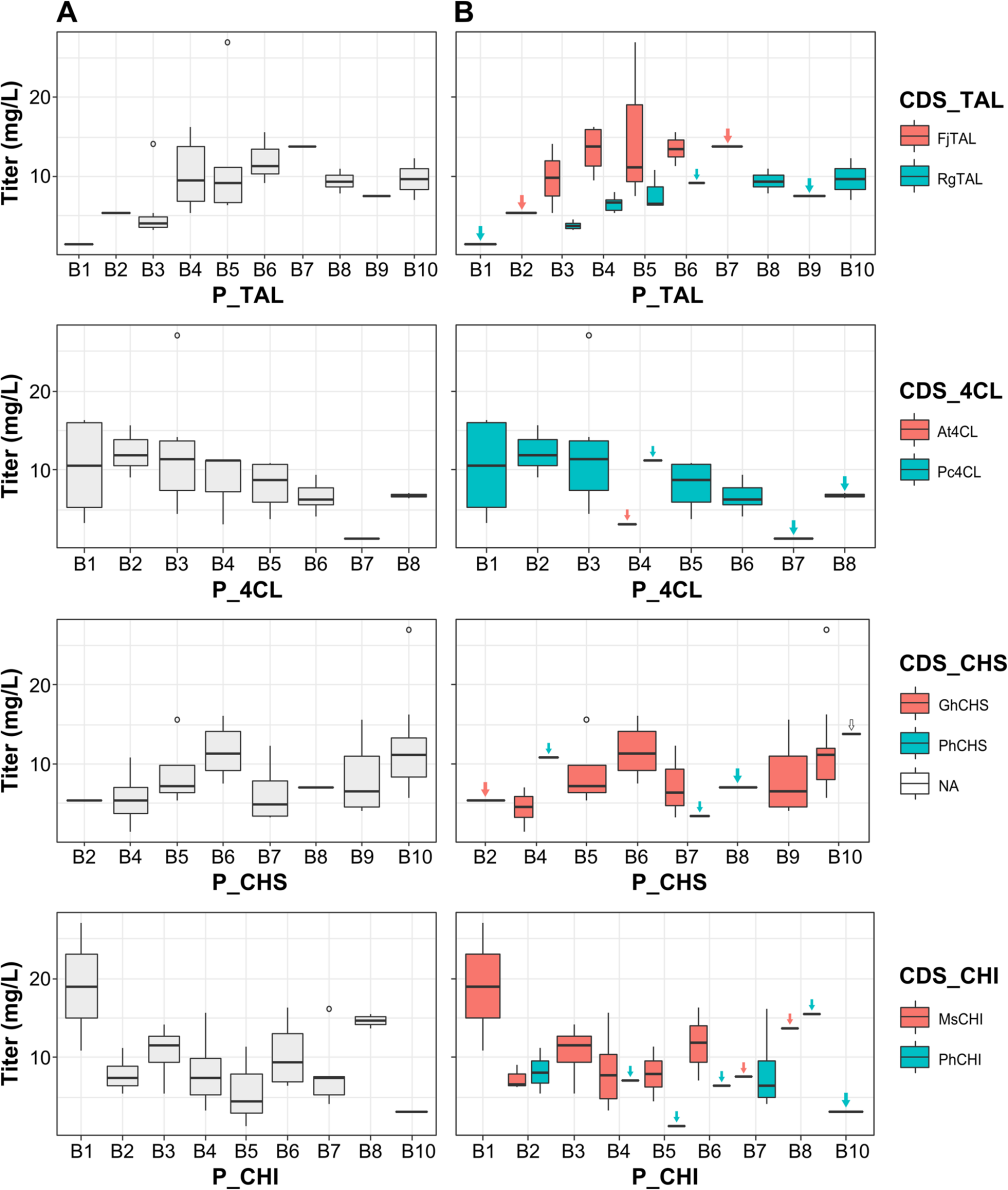
**A** Barplots visualizing the occurrence frequency of each promoter variant (B*X*) at each enzymatic step in the characterized pathways (TAL, 4CL, CHS, CHI) versus the achieved naringenin titer with those pathways containing the specific promoter—enzyme combination. **B** Identical to **A** but each promoter variant occurence versus the achieved titer is plotted separately for its presence in combination with either of the two isozymes. (P_*X*: promoter TIF of enzymatic step *X*; CDS_*X*: enzyme variant; NA: not applicable due to failed CDS DNA sequencing; Rg: *Rhodotorula glutinis*; Fj: *Flavobacterium johnsoniae*; Pc: *Petroselinum crispum*; At: *Arabidopsis thaliana*; Ph: *Petunia hybrida*; Gh: *Gerbera hybrida*; Ms: *Medicago sativa*; TAL: Tyrosine ammonia-lyase; 4CL: 4-coumaroyl-CoA ligase; CHS: Chalcone synthase; CHI: Chalcone isomerase)

To identify key influences of enzyme variant choice on the pathway efficiency, for each enzyme type (CDS_*X*, with *X* = TAL, 4CL, CHS, CHI), barplots were generated that compare the occurrence frequency of either of the two variants in the pathway to the achieved titer (see Fig. 4). Similar, also the relationship between promoter TIF, here considered a categorical variable, and titer at each enzymatic step in the pathway (see Fig. 5A) and separately for promoters in combination with either of the two enzyme variants (see Fig. 5B) is depicted. One-way analysis of variance (ANOVA) shows that pathway configurations containing the TAL CDS from *Flavobacterium johnsoniae* (FjTAL) perform significantly better on average than those containing the TAL CDS from *Rhodotorula glutinis* (RgTAL) (see Fig. 4). The overall outperformance of pathway variants incorporating the FjTAL CDS over their RgTAL CDS containing counterparts is especially visible in Fig. 5B.

What specifically stands out is the presence of only a single 4CL CDS from *Arabidopsis thaliana* (At4CL) and three appearances of the CHS CDS from *Petunia hybrida* (PhCHS) in the dataset, out of 34 observations. By using a unique linker-based Golden Gate assembly workflow for the construction of the pathway variants, the presence of all promoters and coding sequences is evenly distributed in the final pathway library [34, 39]. Therefore, the underrepresentation of At4CL and PhCHS in the selected strains is solely attributed to pathway performance. It was decided accordingly to remove the total of four entries in the dataset containing either of these two enzyme variants to reduce the number of model features, and thus model complexity.

#### Linking pathway features to naringenin production

**Fig. 6:**
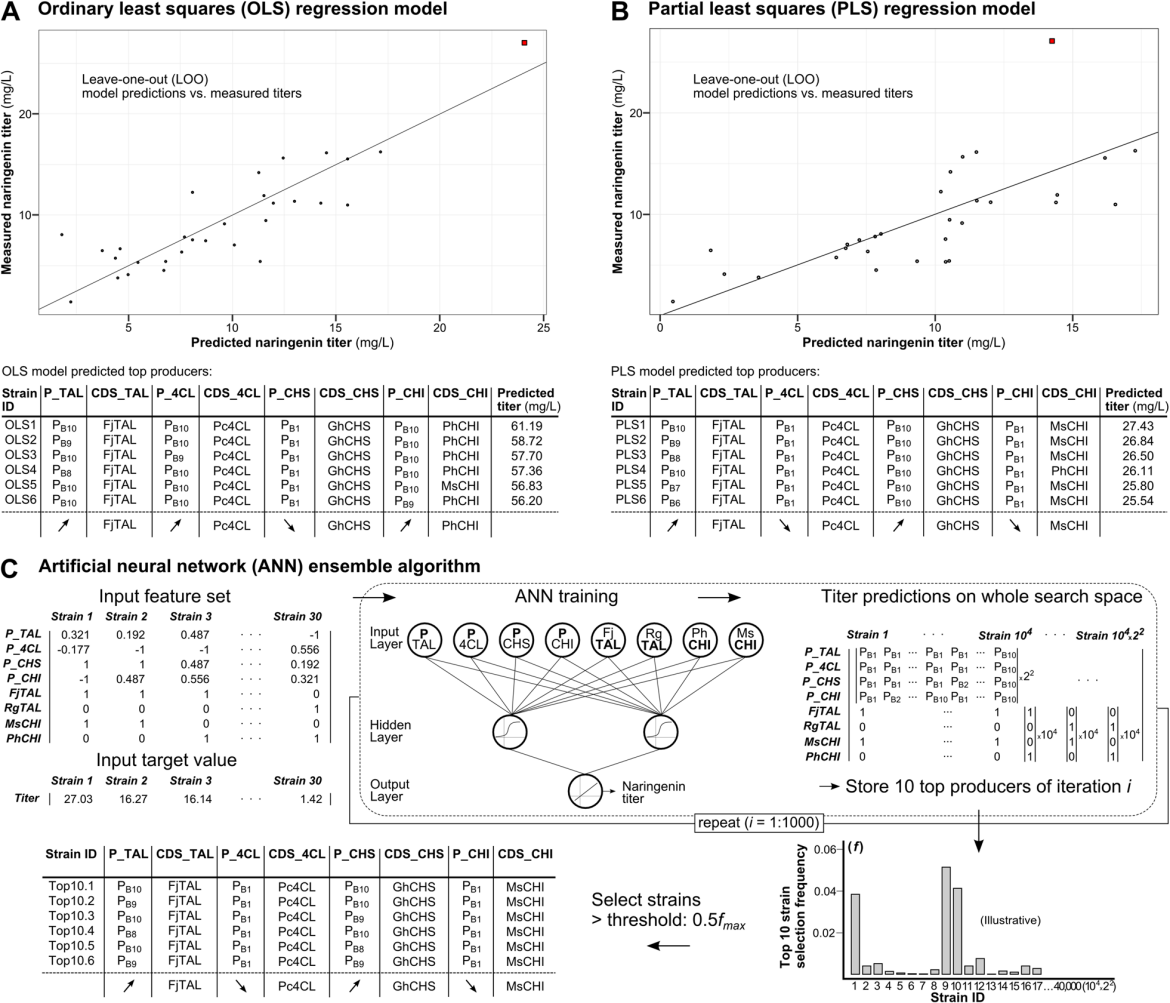
Computer models predicting the optimal pathway architectures to maximize the naringenin production titer in *Escherichia coli*. **A** Ordinary least squares (OLS) regression plot comparing Leave-one-out (LOO) model predictions of the training data to the actual measured titer. The final model holds an R^2^ of 0.93 and p = 1.48*10^–7^ (Additional file 1: Fig. S4). Also the top six predicted producers with pathway architectures and derived consensus architecture are given. **B** Partial least squares (PLS) regression plot comparing LOO model predictions of the training data to the actual measured naringenin production titer. The final model [2] latent variables] explains 78.92% (= R^2^) of the naringenin product titer variance by using 38.82% of the predictors’ variance. Also the top six predicted producers with pathway architectures and derived consensus architecture are given. **C** Machine-learning workflow developed by Zhou et al. [30] to optimize a biosynthetic pathway, here applied for naringenin biosynthesis. 1000 iterations of the ANN (artificial neural network) are trained with random initial weights. In each iteration the titers for the complete search space are predicted and the ten best producers for iteration *i* are stored. The frequency (*f*) of the occurrence of each unique pathway in the Top10 lists of all iterations is calculated and a 0.5*f_*max*_ threshold is set to select the most promising architectures. (P_*X*: promoter driving expression of enzyme *X*; CDS_*X*: enzyme variant of enzyme *X*; Rg: *Rhodotorula glutinis*; Fj: *Flavobacterium johnsoniae*; Pc: *Petroselinum crispum*; Ph: *Petunia hybrida*; Gh: *Gerbera hybrida*; Ms: *Medicago sativa*; TAL: Tyrosine ammonia-lyase; 4CL: 4-coumaroyl-CoA ligase; CHS: Chalcone synthase; CHI: Chalcone isomerase; solid red square: strain 135, top naringenin producer in library screening)

To establish the relationship between the pathway features and the resulting naringenin titer, first an ordinary least squares (OLS) regression analysis was performed, as this method has already proven successful in solving similar biological engineering questions [37, 38, 40, 41]. In the initial model, six features (eight analyzed pathway features, see Fig. 3B, minus CDS_4CL and CDS_CHS) as well as quadratic and interaction terms were included to capture potential non-linear effects that influence product biosynthesis efficiency. Interaction terms between non-matching promoter TIF and CDS features (P_X*CDS_*Y*) and between isozyme features (CDS_X*CDS_*Y*) were excluded because of their assumed biological subordinate relevance. The 30 pathway architectures remaining after the data exploration were used to train the model. This initial model was reduced to only maintain the terms contributing to product formation by sequentially removing the least significant term from the model. The final form and the generated output of the linear regression function in R are displayed in Additional file 1: Fig. S4. An R^2^ measure of 0.93 was obtained. Leave-one-out (LOO) model predictions compared to the measured product titers were plotted and additionally the model was used to predict the production capacity of all possible pathway configurations with the used genetic parts (see Fig. 6A). The predicted top six naringenin producers show a consensus of high FjTAL, *Petroselinum crispum* 4CL (Pc4CL) and PhCHI expression and low *Gerbera hybrida* CHS (GhCHS) expression to reach titers of up to 61.2 mg/L (in the same culture conditions) (see Fig. 6A). This is in contradiction with what could be expected from the performed data exploration for the preferred expression level of 4CL and CHS. On the other hand, the OLS model predicts FjTAL to be the preferred choice over RgTAL, similarly as observed in the data exploration.

As is often the case for predictive modeling for (synthetic) biological engineering, the application’s complexity translates into a large set of predictive features while the experimentally obtainable sample size is relatively small and potentially influenced by the inherent noise on biological systems. Partial least squares (PLS) regression, closely related to principal component analysis (PCA), is developed to cope with these conditions and additionally does not require the assumption of non-multicollinearity [42]. Consequently, this regression method (and PCA) already found its use in diverse applications such as the engineering of metabolic pathways, promoters and RNA devices [43–46]. Therefore, in a second approach a PLS regression model was constructed including the same predictors as the initial OLS model.

The regression model contains 18 regressors and was trained with the 30 observations. Based on model cross-validation (CV) using 30 LOO segments, 2 latent variables (LV) were selected to produce the final model (see Additional file 1: Fig. S5). The model containing 2 LV results in the lowest prediction error and is able to explain 78.92% (R^2^) of the product titer variance by using 38.82% of the predictors’ variance. LOO model predictions result in an R^2^ of 0.5 (see Fig. 6B). The biplot of the 2 LV’s is depicted in Additional file 1: Fig. S6. Also, the regression coefficients of the final model were calculated, indicating the contribution of the predictors to the pathway performance (see Additional file 1: Fig. S7). Again, a major influence of the choice of isozyme for TAL is visible. The regression coefficients explaining promoter TIF all align toward an increased production capacity for high expression of TAL, and in sheer contrast with the OLS model, high expression of CHS and low expression of 4CL and CHI. This is also reflected in the consensus of the top six predicted pathway configurations seen in Fig. 6B. The PLS model predicts naringenin production titers can be achieved of up to 27.43 mg/L naringenin (for the used culture conditions). A visual conception of how the production space is shaped by the complex predictor interactions, is depicted in Additional file 1: Fig. S8. This shows a narrowing production landscape for higher production titers, demonstrating the requirement of smart tools to select the most potent pathways from the gigantic genotypic space.

Recently, Zhou et al. (2018) [30] developed a machine-learning workflow, especially designed to deal with the optimization of heterologous biosynthetic pathways, trained with a relatively small dataset generated from a prescreened combinatorially engineered library, similar to this work. The machine-learning workflow is depicted in Fig. 6C. First, the dataset is reorganized to fit the format accepted by the authors’ custom Matlab script. In the input matrix, promoter TIF is accepted as continuous variable (P_*X*) and every enzyme variant (categorical variables) is given its own input neuron (0: not present in pathway, 1: present). This matrix is used to train 1000 iterations of an artificial neural network (ANN) (architecture: eight input neurons, one hidden layer with two neurons and one output neuron, see Fig. 6C), with random weights assigned to the neural connections. In each iteration, the complete search space is predicted by the trained network and the ten best producers (Top10’s) are stored. Next, a selection threshold of half the frequency of the most occurring pathway architecture in all the stored Top10’s (0.5*f*_max_) is set to select a subset of pathways with the highest production potential (see Fig. 6C). This ANN ensemble workflow (as opposed to the training of a single model) was adopted to avoid overfitting due to the use of a relatively small dataset. In Additional file 1: Table S3, also the Top5 and Top1 selection lists are given, which are obtained similar to the Top10 list, but for this case-study, as opposed to the study by Zhou et al. [30], these lists contain no additional new pathway architectures. The genetic parts predicted to contribute to a superior naringenin production are very similar to the PLS model predictions with minor differences for TAL and CHS promoter TIF (P_TAL and P_CHS, see Fig. 6B and C).

Putting the obtained data-driven results to use, it was chosen to construct the ANN ensemble-predicted outperformers (Top10.1–6 strains, see Fig. 6C) and subject these to extensive characterization.

#### In vivo validation of model-based optimized pathways

**Fig. 7:**
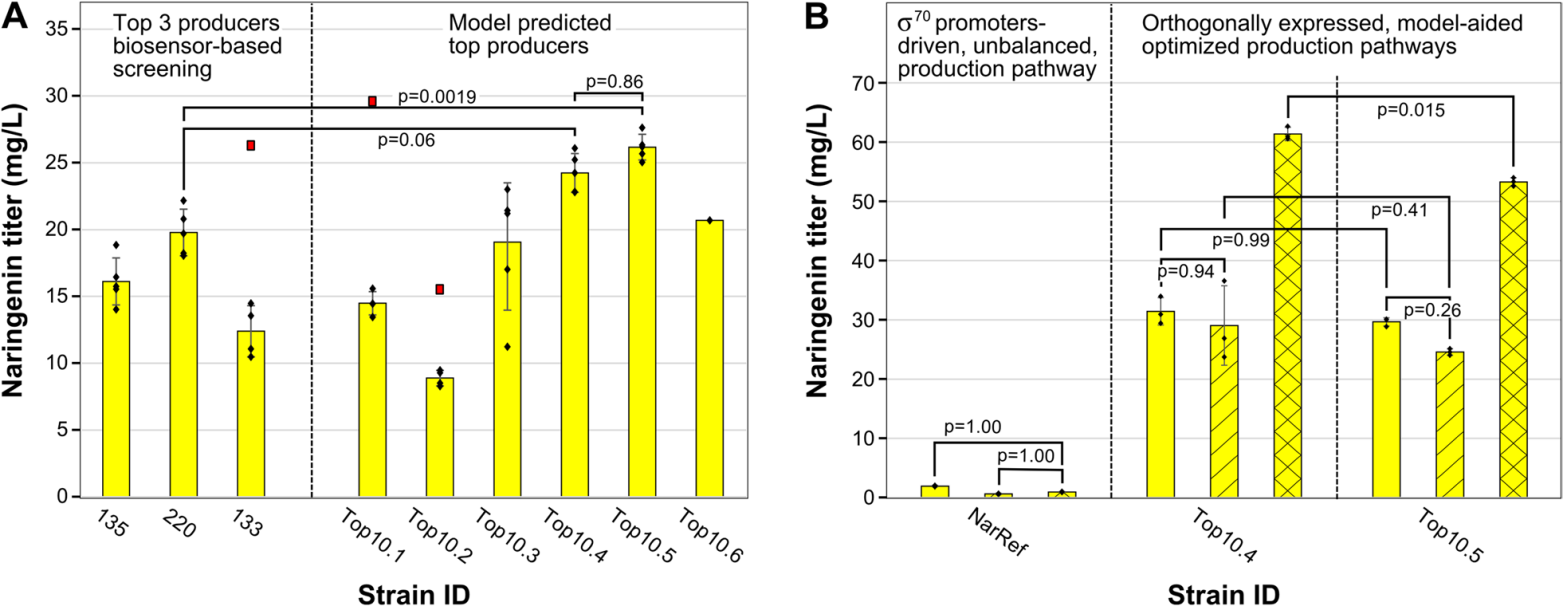
Production titers of: **A** Strains 135, 220 and 133, which were selected as the three best naringenin producers in the biosensor-driven screening (see Fig. 3B) and the six strains, Top10.*x*, bearing the nariningenin biosynthesis pathways which were predicted by the articial neural network (ANN) ensemble model to be the top candidates to yield optimized naringenin production titers (see Fig. 6C). n = 5 except for Top10.6 which has n = 1; **B** The two best performing strains, Top10.4 and Top10.5, compared to a reference strain (NarRef) expressing a non-optimized pathway driven by an identical constitutive sigma factor (σ) 70 promoter for each gene and compared to these strains grown on a culture medium that was supplied with glucose (yellow bars with single hatching) or a carbon-equimolar amount of glycerol (yellow bars with cross hatching). (Error bars: standard deviation; Outliers (filled red squares) are identified with Grubbs’ test for outliers [47], Statistical tests: ANOVA and Tukey for multiple comparison)

To test the accuracy of the in silico predictions of the machine learning workflow, six pathways, Top10.*x* (*x* = 1:6), of which Top10.1, Top10.2 and Top10.4 are identical to the PLS model predicted strains PLS1, PLS2 and PLS3, respectively (see Fig. 6B and C), were constructed for in vivo evaluation. As a reference, the three strains originating from the biosensor-driven product screening holding the highest production titer (see Fig. 3B, strain ID: 135, 220 and 133) are rebuilt, lacking the additional biosensor plasmid (pSynSens1.100) for this purpose. The results are presented in Fig. 7A.

Reference strain 135 produced 27.03 mg/L naringenin (n = 1) in the biosensor-driven screening process, while the same pathway results in, respectively, 16.12 ± 1.76 mg/L (n = 5) in this experiment. Inconsistencies between both observations could be addressed to biological variability or the presence of the naringenin-responsive biosensor plasmid. Due to this difference, the previous observation of strain 135 (see Fig. 3B) is labeled as a biological outlier. The strains bearing the modeled production pathways, Top10.4 and Top10.5 (see Fig. 7A), show a naringenin production titer improvement of respectively 22.5 ± 13.1% and 32.3 ± 12.6% over strain 220, the best producing reference strain. The measured titers of Top10.4 and Top10.5 are 24.24 ± 1.45 and 26.18 ± 0.96 mg/L, which are closely in line with their PLS regression model predictions of 26.50 and 24.53 mg/L, respectively, showing the potential of this model. Conversely, strains Top10.1, Top10.2 and, to some extent, Top10.3 notably underperform compared to the predicted outcome. These results imply that, however high expression for TAL and CHS can be beneficial for production, pathway architectures including expression levels of P_B9_ or higher for both FjTAL and GhCHS combined are detrimental for pathway performance, pointing to metabolic burden, which is also suggested by the occurrence of outliers. Comparing the geno- and phenotypes of Top10.4 with Top10.2 and Top10.5 with Top10.3 (an increase of promoter TIF for either FjTAL or GhCHS, respectively), indicates that the negative effect of metabolic burden is larger for an increase in FjTAL expression, although the actual protein abundance does not necessarily scale equally for different proteins.

Subsequently, the production capacity of the two top producers, Top10.4 and Top10.5, expressing optimized σ^B^-specific-promoters-driven pathways, were benchmarked against an *E. coli* strain bearing an unbalanced naringenin biosynthesis pathway (NarRef). The NarRef pathway is constructed with a combination of CDS variants as used by Santos et al*.* (2011, RgTAL, Pc4CL, PhCHS and MsCHI) [19] and driven by four identical σ^70^ promoters of medium TIF (P22) [48]. Additionally, strains Top10.4 and Top10.5 were also cultured in the same conditions with 1.5% glycerol, instead of 0.1% glucose, supplemented to the growth medium as carbon source because of its reported ability to support the metabolic flux toward both precursor molecules tyrosine [49] and malonyl-CoA [50], of which the latter is a known bottleneck molecule in the flavonoid biosynthesis pathway [24, 51]. To differentiate between the effect on production from glycerol or from the significantly higher carbon source concentration, these strains were also grown on a carbon-equimolar amount of glucose supplied to the medium, replacing glycerol. Cultured on the basic medium, both optimized strains show, on average, an almost 15 times increase in production titer compared to NarRef (see Fig. 7B). The supplementation of glycerol to the medium increases the titer further by 116.8 ± 5.6% to 53.3 mg/L for strain Top10.5 and 111.2 ± 48.9% to 61.4 mg/L for Top10.4, as compared to production on the growth medium containing an equal cmol amount of glucose. Interestingly, while grown on glucose, no significant difference in production titer could be detected between both these strains, if supplemented with glycerol, Top10.4 does perform significantly better than Top10.5 (see Fig. 7B). This suggests that when the malonyl-CoA supply increases, the genotype of Top10.4 (P_TAL < P_CHS) is used more efficiently as compared to the genotype of Top10.5 (P_TAL > P_CHS). Furthermore, when grown on the glycerol supplied medium, the *p*-coumaric acid pool is completely drained, while in abscence of glycerol, some *p*-coumaric acid accumulation is observed (see Additional file 1: Fig. S9). These observations altogether could support the theory that the elevated malonyl-CoA supply boosts the positive effect of high GhCHS expression and at the same time reduces the requirement of the *p*-coumaric acid-accumulation-push-effect, effectuated by the, postulated above, heavily burdensome FjTAL.

### Bioreactor-scale in-depth characterization of the obtained optimal naringenin producing microbial cell factory.

**Fig. 8:**
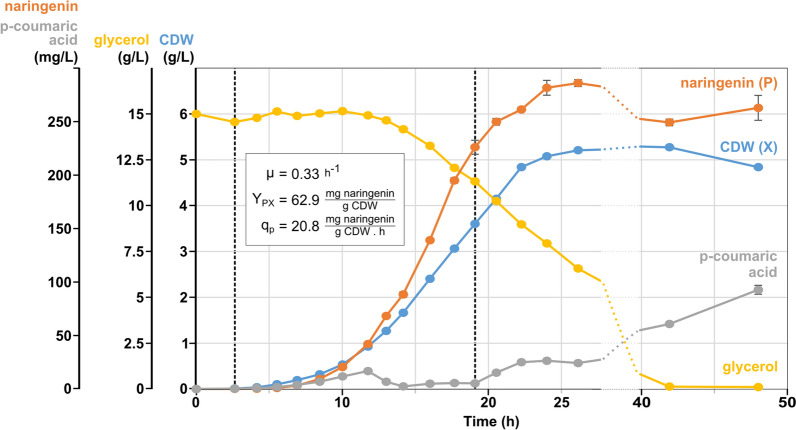
Batch fermentation with production strain Top10.4. The production profiles for cell dry weight (CDW), naringenin and *p*-coumaric acid, and the substrate usage of glycerol is shown. Also, growth rate (µ), yield (Y_PX_) of product (naringenin, P) on biomass (CDW, X) and the specific production rate (q_p_) for naringenin are given for the time frame indicated between dashed vertical lines, encompassing the production process up to the transition to the stationary growth phase. (Error bars = product extraction technical error, n = 2)

The best producing strain, Top10.4 (see Fig. 7), was cultivated in a bioreactor with a working volume of 1.5 L, to characterize its growth and production parameters. Samples were taken throughout the exponential and stationary phase to determine the optical density (~ biomass) and naringenin, *p*-coumaric acid and glycerol concentrations. The resulting profiles in function of the time are given in Fig. 8, and extended with an overview of process parameters in Additional file 1: Fig. S10. Also, the growth rate (µ = 0.33/h), yield of product (P) on biomass (X) formation (Y_PX_ = 62.9 mg P/g CDW) and specific production rate (q_p_ = 20.8 mg P/g CDW/h) for naringenin production are calculated for the time frame indicated in Fig. 8.

When reaching stationary phase, after ± 26 h fermentation time, a titer of 286 ± 3 mg/L (error is standard deviation on product extraction) naringenin was achieved. In the stationary growth phase, no additional naringenin production was observed, though *p*-coumaric acid accumulation started. This could indicate insufficient malonyl-CoA formation, which blocks the pathway upstream of *p*-coumaric acid. However, platings on LB-agar of the first fermentation samples showed a yellow coloration of the agar, the color of naringenin chalcone, while for further platings this effect diminished, and microscopic images of these samples showed filamentation of the cells, which is a sign of stress [52] (see Additional file 1: Fig. S11). Following these observations, the production plasmid of two, non-coloring, single colonies was analyzed by sequencing, showing different large deletions in the pathway genes, which obviously contributes to production termination. Another interesting observation is the remaining glycerol concentration of ± 6 g/L when the stationary phase is reached. This implies that the cells suffer from nutrient depletion, other than the carbon source, after growth to 5 g/L CDW.

## Discussion

Though the progress made in the area of metabolic engineering, and especially synthetic biology, unlocks an enormous potential to boost the biotechnological industry, most MCFs still hit a wall in terms of production performance. The rapidly expanding synthetic biology toolbox supports the emergence of new engineering strategies to breach the current limits of industrial biotechnology, such as the implementation of an orthogonal synthetic regulation network, controlling the metabolic flux through different modules of the pathway [8]. Balancing of the metabolic flux within these orthogonally expressed modules is a prerequisite to guarantee the optimal functioning of these systems as a whole and accordingly, to minimize experimental effort, a general applicable workflow for fast and robust pathway optimization is a necessity. In this study, the optimization of an orthogonally expressed naringenin-producing module was tackled successfully by employing a DNA sequence-independent combinatorial assembly method in combination with a biosensor-driven product screening, followed by the application and assessment of three different computer models to predict optimal pathway architectures. These predictions resulted in the construction of a strain with a 32.3 ± 12.6% increase in production titer as compared to the best producer found in the constructed pathway library.

Evaluating the obtained dataset shows that the pathway library assembly method described by Coussement et al. (2017) [34], complemented in this work with the use of CLIVA for the integration of discrete promoter levels, shows no exclusion of certain library building blocks in the assembly, thereby allowing a broad coverage of the defined search space (see Fig. 4 and Fig. 5). Moreover, due to the use of DNA sequence independent assembly methods, any underrepresentation of certain genetic parts among the selected strains would be solely attributable to its biological underperformance for product biosynthesis. This potentially allows the rational reduction of the model complexity by dropping these feature levels (or features), as is the case in this study for At4CL and PhCHS (features CDS_4CL and CDS_CHS).

With our dataset it was possible to train an OLS and PLS regression model and the ANN ensemble developed by Zhou et al. (2018) [30], thereby obtaining an R^2^ of 0.93 and 0.79, respectively, for the OLS regression model and the PLS model with two latent variables. Although the OLS regression model holds a relatively high R^2^, its predictions are inconsistent with the exploratory data analysis and the PLS and ANN ensemble predictions. On top of that, the naringenin production titer of strain 135, which could, after further characterization, be labeled as an outlier in the dataset, is predicted closely to its measured value by the OLS regression model, even if the observation is not included for model training (see LOO cross validation predictions, Fig. 6A). This suggests that the OLS model building process (dropping the non-significant features from the model) is heavily influenced by outliers, resulting in the elimination of potentially important pathway features in the final model. Indeed, when the OLS model building procedure is repeated after exclusion of strain 135, the predictions align more closely to the PLS and ANN ensemble results (data not shown). This surfacing issue is most likely caused by overfitting due to the relatively small dataset size, as compared to the number of model features. In these conditions, the PLS regression model and ANN ensemble showed much more robust predictions. Further, by extending the dataset with the six new observations (Top10.*x*), and updating the production titers for strains 135, 220 and 133 after reanalysis, no new potentially better performing strains are identified by either of the two models.

The integrated biosensor-driven modeling approach allowed to maximize the pathway performance in a single DBTL cycle, and this with a dataset comprising as few as 0.02% of all possible pathway architectures with the used genetic parts. To achieve this, obtaining a high quality dataset is key, and the naringenin-responsive biosensor has proven very valuable in this respect. Although the biosensor fails to provide an accurate prediction of the achieved naringenin titers in these conditions, it enabled a fast and easy selection of varying, and all producing, phenotypes. Tuning the biosensor to a more suitable operational range with a lower error could improve the reliability of fluorescence-to-production titer conversion [36], ideally rendering subsequent UPLC analysis unnecessary.

The used pathway optimization approach showed that the pathway performance benefits from high TAL (with a clear preference for FjTAL) and CHS expression while the opposite is true for 4CL and CHI. However, evaluating the pathway architectures which include the strongest promoters to drive both TAL and CHS expression indicated that metabolic burden caused a strong decline in production titer for these expression levels (Top10.1–3 versus Top10.4–6), thus defining the optimization limit with the present genetic building blocks. In this respect, RBS engineering could avert metabolic burden to some extent by improving the economy of gene expression (53, 54). Cell fitness could be enhanced, while still achieving the same protein levels, by combining weaker promoters with higher translation initiation rates, and thus, RBS strength is an interesting feature to add to the workflow.

Beside pathway balancing, addressing limiting precursor pools can be equally important. In the flavonoid pathway, the low basal level of malonyl-CoA has proven a major bottleneck [24, 51]. Consequently, great attention has been given to finding metabolic engineering targets to increase these levels [24, 55–57]. These efforts show that the achieved naringenin production could easily be improved by implementing the gene deletions or knock-downs corresponding to metabolic reactions which consume the required precursor molecules. Additionally, specific genes have been identified which contribute to an elevated precursor pool when overexpressed. These genes are interesting targets for the construction of a second expression module for further optimization. In the past five years, precursor supplementation and/or precursor pathway engineering have generated unprecedented titers for naringenin-producing *E. coli* microbial cell factories. Titers of up to almost 600 mg/L naringenin were achieved depending on the specific precursor engineering strategy factories [58–61]. This clearly demonstrates the potential of complementing this research, which specifically optimized the heterologous naringenin pathway up to high naringenin titers in *E. coli* without any precursor pool engineering, with such precursor engineering strategies.

Also, process and medium optimization is an important step in the development of a MCF. We demonstrate that, by adding glycerol as the sole carbon source, the flux toward malonyl-CoA is sufficiently enhanced to double naringenin production up to 61.4 ± 1.1 mg/L (see Fig. 7). The influence of medium composition and process conditions are still too often overlooked and postponed to the scale-up phase for valorization. Furthermore, comparing our two best producers, Top10.4 and Top10.5, grown on either the basic medium or the glycerol supplied medium shows that different genotypes respond differently to changing medium conditions, which adds a major complication to pathway engineering prior to medium optimization. However, the effect of medium and metabolic precursor engineering on product biosynthesis is also hard to predict if the downstream pathway is not yet engineered. Since including process, medium, precursor and heterologous pathway optimization simultaneously in a single workflow would be very laborious and time-consuming, the use of genetic circuitry could offer a solution by enhancing the flexibility of the engineered strain, adapting expression profiles according to changing extra- and intracellular conditions.

In this study, naringenin production was also scaled to 1.5 L in a bioreactor, thereby achieving a production titer of 286 ± 3 mg/L (technical error) in 26 h. After reaching stationary phase, no increase in production was observed. Since the production profile closely aligns with growth, a fed-batch fermentation could further enhance production titer, although genetic instability issues arise after multiple generations, which could hamper the benefits of a prolonged fermentation. It would be interesting to further research genetic robustness in order to try to alleviate its effects.

## Conlusions

In this study, using our developed orthogonal expression toolset from previous work [8], a very competitive production titer for naringenin is achieved, and this without any precursor supplementation or strain engineering for precursor pool optimization. More specifically, a high-throughput combinatorial pathway library screening process, to obtain a high quality dataset, was combined with predictive modeling by training an OLS, PLS and ANN ensemble model. Here, the PLS and ANN ensemble models clearly outperformed the OLS model. Moreover, the ANN ensemble has proven its value as a perfect, easy-to-implement alternative to more established regression methods, requires no prior knowledge for a rational selection of relevant higher order/interaction terms and is designed to deal with (small dataset-related) overfitting issues. The complete workflow could contribute to any pathway optimization process for the production of various industrially relevant compounds, in case there is a high-throughput screening method available. Lastly, although the performance of the heterologous naringenin biosynthesis pathway was successfully improved, it is important not to overlook the potential significance of medium and fermentation process conditions differently affecting different genotypes.

## Materials and methods

### Media, strains and plasmid construction

All products were purchased from Sigma-Aldrich (Diegem, Belgium) unless otherwise stated. Agarose and ethidium bromide were purchased from Thermo Fisher Scientific (Erembodegem, Belgium). Standard molecular biology procedures were conducted as described by Sambrook et al. (1989) [62]. All DNA fragments were amplified using PrimeSTAR HS DNA polymerase (Takara, Westburg, Leusden, The Netherlands) and purified using the innuPREP PCRpure Kit (Analytik Jena AG, Jena, Germany).

Lysogeny broth (LB) was used for cloning purposes. Complex medium (853) was used for all further experiments, with small modification for the glycerol supplied medium. LB medium was composed of 10 g bacto-tryptone, 5 g yeast extract and 5 g NaCl in 1 L water. 853 medium was composed of 10 g bacto-tryptone, 5 g yeast extract, 1 g glucose, 5 g NaCl, 0.7 g K_2_HPO_4_ and 0.3 g KH_2_PO_4_ in 1 L water. For the glycerol supplied medium, 1 g/L glucose was replaced with 15 g/L glycerol. The relevant antibiotics were added to the media, kanamycin (50 µg/mL), chloramphenicol (25 µg/mL) and ampicillin (100 µg/mL).

*E. coli* Top10 cells (Invitrogen, Carlsbad, U.S.A.) were used for cloning purposes. The *E. coli* MG1655 strain bearing the heterologous σ^B^ in the genome [8] was used for all further experiments requiring production pathway expression. An overview of the different used plasmid backbones and their purpose in this study are listed in Table 1. The plasmid carrying the naringenin-responsive biosensor, created by De Paepe et al. (2018) [36], and all carrier and expression vectors, created by Coussement et al*.* (2017) [34], which were used for pathway (library) cloning, were available in the lab.

**Table 1:** Overview of the different plasmid backbnes used in this study and their assigned function

Plasmid	Use in study	Copy number	Antibiotic	References
pUC	Donor vector pathway assembly	~ 500–700	Amp	[34]
pBR322	Pathway expression vector	~ 15–20	Kan	[34]
pSC101	pSynSens1.00 – naringenin-responsive biosensor	~ 5	Chlor	[36]

*Amp* ampicillin, *Kan* kanamycin, *Chlor* chloramphenicol

For the followed pathway construction workflow, all enzyme variants in the pathway (listed in Table 2) were cloned in separate carrier plasmids, in which all operons catalyzing the same enzymatic reaction are flanked by the same pair of Golden Gate (GG) restriction sites, sequentially matching with the GG restriction sites for the different steps in the pathway [34] (see Fig. 2). Therefore, promoterless CDSs were cloned in the carrier vectors using Circular Polymerase Extension Cloning (CPEC) after which the promoter (-libraries) were inserted in a 2-piece CLIVA reaction (35). After the construction of the vectors containing libraries, the complete transformation mixture was incubated in fresh medium for subsequent plasmid extraction. The relevant DNA sequences were verified by Sanger sequencing service (Macrogen Inc., Amsterdam, The Netherlands).

**Table 2:** Used enzymes for the construction of the naringenin biosynthesis pathway (library)

Abbrev	Organism of origin	Function	EC	Source	References
RgTAL	*Rhodotorula glutinis*	Tyrosine ammonia-lyase	4.3.1.23	(19)	[19, 22, 63–65]
FjTAL	*Flavobacterium johnsoniae*			This study	[66]
Pc4CL	*Petroselinum crispum*	4-coumaroyl-CoA ligase	6.2.1.12	(19)	[19, 22, 63, 67, 68]
At4CL	*Arabidopsis thaliana*			iGEM2014:BBa_K1497016	[21]
PhCHS	*Petunia hybrida*	Chalcone synthase	2.3.1.74	(19)	[19, 22, 63, 67, 68]
GhCHS	*Gerbera hybrida*			iGEM2014:BBa_K1497016	[69]
MsCHI	*Medicago sativa*	Chalcone isomerase	5.5.1.6	(19)	[19, 22, 63, 68]
PhCHI	*Petunia hybrida*			iGEM2014:BBa_K1497016	[67, 68]

In addition, their organism origin, function, enzyme classification numbers (EC), source and references are given. The DNA sequence of FjTAL, synthesized in this study, is given in Additional file 1: Table S2.

Subsequently, all pathway (library) fragments were put together in the expression vector backbone, in a one-pot, 5-piece GG reaction. The complete annotated nucleotide sequence (genbank format) of the assembled naringenin biosynthesis pathway Top10.4 is given in Additional file 1: Fig. S12, which is representative for all pathway variants only differing in promoter [8] and CDS (see Table 2).

### Library screening and characterization

For the fluorescence (FL) based library screening, freshly made electrocompetent *E. coli* MG1655 cells were first transformed with pSynSens1.100, after which cells were made competent again for electroporation with the GG assembly mix containing the pathway variants.

Library screening was performed by randomly picking single colonies by hand after transformation, and incubation in 150 µL 853 medium in sterile 96-well flat-bottomed black MTPs (Greiner Bio-One, Vilvoorde, Belgium), enclosed by a Breath-Easy^®^ sealing membrane (Sigma-Aldrich) for 24 h at 30 °C while shaking (800 rpm in a Compact Digital Microplate Shaker, ThermoFisher Scientific). The optical density at 600 nm (OD_600_) and biosensor produced FL was measured (mKate2, excitation: 588 nm and emission: 633 nm) in a Tecan Infinite M200 Pro plate reader. The reported values were obtained by first correcting FL and OD_600_ for growth medium (blank) and subsequently, calculating the FL over OD_600_ ratio:

2 $$\left( {\frac{FL}{{OD_{600} }}} \right)_{corrected } = \frac{{FL - FL_{blank} }}{{OD_{600} - OD_{600, blank} }}$$

The biosensor was also characterized on its own (*E. coli* MG1655 + pSynSens1.100), simultaneously with the screening process and in similar manner, with addition of the indicated naringenin concentrations to the medium.

To determine the genotype of the 35 fluorescence-based selected library strains, first, cells from the MTP cultures were streaked on agar plates lacking the antibiotic required to sustain replication of the biosensor carrying plasmid. Next, single colonies were cultured for plasmid isolation and subsequent Sanger sequencing (Macrogen Inc., Amsterdam, The Netherlands). Sequence alignment was used to determine the pathway architectures.

To prepare the samples for product quantification after incubation, 100 µL of the MTP cultures was transferred to 1.5 mL tubes and product was extracted with double volume of ethyl acetate by vigorous shaking for 3 min at 1600 rpm in a BioShake iQ (QInstruments) shaker. Subsequently, the organic layer was isolated and evaporated to dryness. The remaining products were dissolved in ethanol for UPLC-UV analysis.

### In vivo model validation

*E. coli* MG1655 cells were transformed with the plasmids containing the pathway architectures from strain 135, 220, 133 and Top10.1–6, and subsequently cultured, analogous as performed for the pathway library strains. After incubation, 100 µL of the MTP cultures was transferred to 1.5 mL tubes, and samples were prepared for naringenin quantification, also as described above.

### Bioreactor scale production

For in-depth characterization of production strain Top10.4, a batch bioreactor experiment was set up using a Biostat B + reactor (Sartorius Stedim, Germany) with a working volume of 1.5 L glycerol supplied 853 medium. Prior to inoculation, the process parameters were set at an airflow rate of 1 vvm, 600 rpm stirrer speed, 30 °C and pH 7.0. Also 1 drop of antifoam agent (STRUKTOL® J 673, Schill + Seilacher) was added. The pO_2_ electrode was calibrated with 0% indicating a zero signal and stirrer speed was temporary raised to 1000 rpm to set the 100% level.

The reactor was inoculated for 1% of the medium volume with a freshly transformed and exponentially growing preculture (853 medium). During fermentation, the culture temperature was maintained at 30 °C and 5 M KOH and 0.5 M H_2_SO_4_ solutions were automatically added to keep the pH at 7.0. All parameters were monitored and adjusted if necessary with MFCS/win software (Sartorius AG).

During the fermentation, samples were taken regularly for OD_600_ and metabolite analysis. The OD_600_ was measured with a Jasco V-630Bio spectrophotometer (Easton, UK) and 1 mL supernatant and 1 mL broth were stored at  − 20 °C for further analysis.

The conversion of OD_600_ to cell dry weight (CDW) was determined by pelleting 20 mL of the final fermentation broth, washing with physiological solution, and drying the pellets for 24 h at 70° C before weighing. The determined OD_600_ to CDW conversion is described as:

3 $$CDW = OD_{600} *0.29$$

Deviating from the sample preparation for product quantification of the MTP-scale cultures, ethanol extraction was used because of its outperforming product recovery efficiency for both *p*-coumaric acid and naringenin, which was revealed after further method optimization. An equal volume of ethanol was added to the fermentation broth samples and the mixture was vigorously shaken for 3 min at 1600 rpm in a BioShake iQ (QInstruments) shaker. Cell debris was removed by centrifugation and the 50% ethanol mixture was used for UPLC-UV analysis. The unprocessed fermentation broth supernatant was used for HPLC-RI glycerol quantification.

Fermentation parameters were calculated as follows, and in the time frame indicated in Fig. 8. Growth speed (µ) is the slope of the natural logarithm transformed growth curve, determined by linear regression, as:

4 $$X = X_{0} *e^{\mu *t}$$

with X the biomass, X_0_ the initial biomass and t the time. The yield (Y) of product (P) on biomass (X) is calculated as:

5 $$Y_{PX} = \frac{\Delta P}{{\Delta X^{^{\prime}} }}$$

in the given time frame.

The specific production rate (q_p_) is calculated as:

6 $${\text{q}}_{{\text{p}}} = \upmu * {\text{Y}}_{{PX}}$$

### Analytic methods

Prior to analysis, all samples were filtered through a PTFE filter (VWR, Leuven, Belgium). Naringenin and *p*-coumaric acid were quantified using a Waters Acquity UPLC H-Class system connected to an ACQUITY TUV-detector operating at 30 °C and 290 nm. A Kinetex^®^ 2.6 µm Polar C18 100 Å column (Phenomenex, Utrecht, The Netherlands) was used to separate metabolites using the following method, at a flow rate of 0.6 mL/min:

**Table:** 

Time (min)	Eluent A: 0.1% TFA in water (%)	Eluent B: 100% acetonitrile (%)
0	90	10
0.5	75	25
5	75	25
7	30	70
8.5	30	70
10	90	10

Glycerol was quantified on a Shimadzu Prominence-I LC2030c Plus system connected to an RID-20A (Shimadzu) detector operating at 40 °C. A Rezex ROA-Organic Acid H + (8%) – 150 × 7,8 mm column (Phenomenex, Utrecht, The Netherlands) at 60 °C was used to separate metabolites using an isocratic method with a flow rate of 0.6 mL/min and 0.005 N H_2_SO_4_ in water as eluens.

### Statistical methods, regression models and machine learning

All data processing, statistical testing and modeling was performed with a custom written R script except for Grubbs’ test for outliers [47], which was implemented manually in Microsoft® Office Excel. For all significance testing between means, Analysis of Variance (ANOVA) was used, followed by Tukey’s honest significance test for the comparison of multiple means, if applicable. For the data depicted in Fig. 7, all means were included in Tukey’s test though only the relevant p-values are shown. Error bars depict standard deviation of biological replicates, unless stated otherwise.

The obtained dataset from the biosensor-driven screening process was preprocessed before any statistical testing or model building. Data for one strain was removed due to a missing value and all promoter TIF data was linlog transformed according to:

7 $$X = \frac{{\log \left( P \right) - \frac{{\log \left( {P_{\max } } \right) + \log \left( {P_{\min } } \right)}}{2}}}{{\frac{{\log \left( {P_{\max } } \right) - \log \left( {P_{\min } } \right)}}{2}}},$$

with *X* the transformed data, *P*_*min*_ the value of P_B1_ and *P*_*max*_ the value of P_B10_ (see depicted in Additional file 1: Fig. S2).

#### Regression models

##### Ordinary least squares (OLS) regression

Equation 8 depicts the linear relationship established by the OLS regression, where Titer_i_ is the obtained titer in mg/L for pathway i, P_X,i_ and CDS_X,i_ are the promoter- and enzyme variants of enzyme X for pathway i, β_1-18_ are the regression coefficients and ε_i_ an error term. For the categorical pathway features (CDS_X_), the default 0/1 dummy coding is used describing either of two enzyme variants.

8 $$\begin{gathered} Titer_{i} = {\upbeta }_{0} + {\upbeta }_{1} P_{TAL,i} + {\upbeta }_{2} P_{4CL,i} + {\upbeta }_{3} P_{CHS,i} + {\upbeta }_{4} P_{CHI,i} \hfill \\ + {\upbeta }_{5} CDS_{TAL,i} + {\upbeta }_{6} CDS_{CHI,i} + {\upbeta }_{7} P_{TAL,i}^{2} + {\upbeta }_{8} P_{4CL,i}^{2} + {\upbeta }_{9} P_{CHS,i}^{2} \hfill \\ + {\upbeta }_{10} P_{CHI,i}^{2} + {\upbeta }_{11} P_{TAL,i} CDS_{TAL,i} + {\upbeta }_{12} P_{CHI,i} CDS_{CHI,i} \hfill \\ + {\upbeta }_{13} P_{TAL,i} P_{4CL,i} + {\upbeta }_{14} P_{TAL,i} P_{CHS,i} + {\upbeta }_{15} P_{TAL,i} P_{CHI,i} \hfill \\ + {\upbeta }_{16} P_{4CL,i} P_{CHS,i} + {\upbeta }_{17} P_{4CL,i} P_{CHI,i} + {\upbeta }_{18} P_{CHS,i} P_{CHI,i} + {\upvarepsilon }_{i} \hfill \\ \end{gathered}$$

where Eq. 8 depicts the initial OLS regression model, the same equation leaving out the highlighted terms gives the final model. These terms were sequentially removed from the model based on their insignificant contribution (highest p-value) to the model performance. Lower order model terms were not dropped from the model if any higher order terms including the feature were still in. The model was considered final for an R^2^ > 0.9 and p-value < 0.15 (see Additional file 1: Fig. S4).

##### Partial least squares (PLS) regression

For PLS regression, the R pls package was used [70]. The regressors from the OLS model, depicted in Eq. 8, were reused for PLS regression. The linear relationship shown in Eq. 8 can be written in its general form as described in Eq. 9, with **Y** representing a matrix containing the production titers, **X** a matrix with the input variables, **B** the matrix with regression coefficients and **ε** the error matrix. In PLS regression, the matrix of predictors **X** is decomposed into orthogonal score matrix **T** (projection of **X**) and loadings matrix **P.** Next, **Y** is not regressed on **X** but on the first *a* rows of score matrix **T**, with *a* the number of latent variables kept in the model.

9 $$\begin{gathered} Y = {\text{X B}}\,{ + }\,{\upvarepsilon } \hfill \\ {\text{X}}\,{ = }\,{\text{T P}} \hfill \\ \end{gathered}$$

For both OLS and PLS, leave-one-out cross validation was used, where the model is trained *n* times, leaving out one observation at a time after which the output for the left out datapoint is predicted, and this for a total of *n* datapoints.

#### Machine learning

For pathway optimization through machine learning, the workflow and model described by Zhou et al. (2018) [30] was adopted. The artificial neural network structure is composed of 3 layers, an input layer with a neuron for each input pathway variable, a hidden layer with two neurons and one output neuron for production titer. For this work, all levels of categorical variables (= CDS variants) were given its own input neuron. The Levenberg–Marquardt backpropagation function was used to train the network in maximum 100 cycles, at a learning rate of 0.01. A log-sigmoid activation function connects the neurons of the input layer with the neurons of the hidden layer and the connection to the output neuron is established via the linear activation function. The training-prediction procedure was repeated 1000 times. In each iteration the ten best predicted producers are stored and after the last iteration, for every different strain in those lists, the frequency (*f*) of their occurrence is calculated. Subsequently, a threshold of 0.5*f*_*max*_ is set to make a subselection of the most promising producers. The ANN model and selection procedure was performed as described by Zhou et al. [30].

## Supplementary Information

**Additional file 1: Table S1.** Pathway architecture of the strains selected through the biosensor-driven combinatorial engineering process. The naringenin titer determined by UPLC analysis and the biosensor output is also given for each strain (n = 1). (TAL: Tyrosine ammonia-lyase; 4CL: 4-coumaroyl-CoA ligase; CHS: Chalcone synthase; CHI: Chalcone isomerase; Nar.: naringenin; Fluo: fluorescence; a.u.: arbitrary units CDS: coding DNA sequence; NA: not applicable, failed sequencing). **Table S2.** NUCLEOTIDE SEQUENCE OF THE CODON OPTIMIZED FJTAL, NEWLY SYNTHESIZED FOR THIS STUDY. **Table S3.** output of the artificial neural network (ANN) ensemble in the format described by Zhou *et al.* [30]. Pathway configurations are included in the Top *x* list if, after 1000 ANN train and predict iterations, the frequency of their occurrence in the predicted top *x* strains is higher than half the frequency of the most occurring strain in that top list ( f(top *x*) > 0.5*f_max_(top *x*) ). Identical colors are used to indicate identical strains. (P_*X*: promoter variant of enzymatic step *X*; CDS_*X*: enzyme variant; freq: frequency (f)). **Figure. S1** Characteristics of the naringenin-responsive biosensor (pSynSens1.100 [36]) in the conditions used in this study. (**A**) The responsive curve and fitted Hill function for a supplied naringenin concentration range of 0-100 mg/L and the corresponding Hill parameters. Also the operational range and Noise parameter are given, as determined with the method described by De Paepe et al. (2018) [36] and depicted in (**B**). (a: the basal normalized fluorescent signal (a.u., arbitrary units); M: the maximum normalized fluorescent signal (a.u.); n: Hill coefficient (cooperativity); K: Hill constant (transcription factor – ligand affinity, mg/L); error bars: standard errors for 5 biological replicates, n = 5). **Figure. S2** Linlog transformation of the sigma B promoter library promoters as input for the created models. (**A**) Original data, displayed as sfGFP corrected mKate values [8]. (**B**) Linlog transformed promoters. (**C**) Used linlog transformation and properties of the transformed data. (TIF: transcription initiation frequency; P = untransformed promoter TIF, X = linlog transformed promoter TIF). **Figure. S3** Correlation (Pearson, ρ) between the continuous variables (promoter transcription initiation frequency and titer) in the dataset, shown as the generated output of the corr.test() function of the R psych package [71]. The top matrix shows the correlation between the variables with -1 and 1 indicating a perfect (inverse) correlation and 0, no correlation. The bottom matrix shows the corresponding probability values (Null hypothesis = ‘H_0_: 2 variables are not correlated (ρ = 0)’). The Holm-Bonferroni method is used to adjust for multiple testing. (Rg: *Rhodotorula glutinis*; Fj: *Flavobacterium johnsoniae*; Pc: *Petroselinum crispum*; At: *Arabidopsis thaliana*; Ph: *Petunia hybrida*; Gh: *Gerbera hybrida*; Ms: *Medicago sativa*; TAL: Tyrosine ammonia-lyase; 4CL: 4-coumaroyl-CoA ligase; CHS: Chalcone synthase; CHI: Chalcone isomerase).** Figure. S4** Ordinary least squares regression output from the lm() function in R. The input formula is obtained by a limitted sequential removal of terms holding the highest p-value, starting from the full quadratic regression model. (Q: quadrant; P_*X*: promoter transcription initiation frequency for expression of enzyme *X*; CDS_X: coding sequence variant of enzyme *X*; P_*X*:P_*X*: interaction term; I(P_*X*^2): quadratic term; Rg: *Rhodotorula glutinis*; Fj: *Flavobacterium johnsoniae*; Pc: *Petroselinum crispum*; At: *Arabidopsis thaliana*; Ph: *Petunia hybrida*; Gh: *Gerbera hybrida*; Ms: *Medicago sativa*; TAL: Tyrosine ammonia-lyase; 4CL: 4-coumaroyl-CoA ligase; CHS: Chalcone synthase; CHI: Chalcone isomerase). **Figure. S5** Cross-validated (CV) root mean squared error of prediction (RMSEP) curve. A model only including the first two components (*i.e.* latent variables, LV) shows the lowest prediction error. A model with two LV predicts 78.92% of the product titer by using 38.82% of the predictors’ variance. (adjCV: adjusted CV). **Figure. S6** Biplot of the first two components of the partial least squares (PLS) regression model. (P_*X*: promoter transcription initiation frequency (TIF) of enzymatic step *X*; CDS_*X*: enzyme variant; I(P_*X*^2): quadratic term of promoter transcription initiation frequency; ‘P_*X*:CDS_*X*’ and ‘P_*X*: P_*Y*’: promoter TIF interaction terms with enzyme variants or between the promoter TIFs of two different enzymatic pathway reaction steps; TAL: Tyrosine ammonia-lyase; 4CL: 4-coumaroyl-CoA ligase; CHS: Chalcone synthase; CHI: Chalcone isomerase). **Figure. S7** The estimated partial least squares (PLS) regression coefficients of all pathway features, including quadratic and interaction terms. (P_*X*: promoter transcription initiation frequency (TIF) of enzymatic step *X*; CDS_*X*: enzyme variant; I(P_*X*^2): quadratic term of promoter TIF; ‘P_*X*:CDS_*X*’ and ‘P_*X*: P_*Y*’: promoter TIF interaction terms with enzyme variants or between the promoter TIFs of two different enzymatic pathway reaction steps; TAL: Tyrosine ammonia-lyase; 4CL: 4-coumaroyl-CoA ligase; CHS: Chalcone synthase; CHI: Chalcone isomerase). **Figure. S8**: Cross sections of the multidimensional production landscape, predicted by the partial least squares (PLS) model. For each cross section, two pathway features (promoter transcription initiation frequencies, TIF) are varied while the remaining part of the pathway configuration is fixed. The fixed input values, other than the two variables depicted on the x- and y-axes, are set according to the predicted optimal producer (see legend, P_*X*: promoter TIF of enzymatic step *X*; CDS_*X*: enzyme variant; TAL: Tyrosine ammonia-lyase; 4CL: 4-coumaroyl-CoA ligase; CHS: Chalcone synthase; CHI: Chalcone isomerase). **Figure. S9** The UPLC-UV chromatogram of one of the ethyl acetate-extracted biological replications of NarRef, Top10.4, Top10.5 and Top10.4 grown on the glycerol supplied medium. The Top10.5 + glycerol profile is similar to the Top10.4 + glycerol profile and the depicted profiles are also representative for the other biological replications, but are left out for visual clarity. As a reference, also the chromatographic profile of the strain bearing only heterologous sigma factor (σ) B in the genome but no plasmid is included. **Figure. S10** Batch fermentation with production strain Top10.4. In the upper part the production profiles of cell dry weight (CDW), naringenin and *p*-coumaric acid, and the substrate usage of glycerol are given. In the lower part, the process parameter profiles for base and acid addition and dissolved oxygen (PO_2_) are given, together with events of process parameter(-change) indications for airflow, stirrer speed, temperature and antifoam addition. Stirrer speed spikes were used to break accumulated foam. **Figure. S11** (**A**) Plating of the first sample taken of the batch fermentation with production strain Top10.4, compared to an empty LB-agar plate. The yellow coloration is found to indicate product formation, most likely coming from intermediate metabolite naringenin chalcone. (**B**) Gram-stained sample of the performed batch fermentation with strain Top10.4. Filamentation of the production organism is an indication for stress [52]. **Figure. S12** Annotated genbank file of the optimized naringenin biosynthesis pathway (pTop10.4, Figure 6). The expression vector originates from Coussement et al. (2017) [34] (Table 1). The promoters driving the pathway are created in Bervoets et al. (2018) [8]. More information about the enzymes and source of CDSs can be found in Table S2. The used transcription terminators are from the BIOFAB collection [72].

## Data Availability

All data generated or analysed during this study are included in this published article and its Additional file 1: information files.
