# Supplementary material for: Biosensor-driven, model-based optimization of the orthogonally expressed naringenin biosynthesis pathway
Source: Microb Cell Fact. 2022 Mar 27;21:49. doi: 10.1186/s12934-022-01775-8 (PMC8962593; doi:10.1186/s12934-022-01775-8)
Supplement: Supplementary file 1 — Additional file 1: Table S1. Pathway architecture of the strains selected through the biosensor-driven combinatorial engineering process. The naringenin titer determined by UPLC analysis and the biosensor output is also given for each strain (n = 1). (TAL: Tyrosine ammonia-lyase; 4CL: 4-coumaroyl-CoA ligase; CHS: Chalcone synthase; CHI: Chalcone isomerase; Nar.: naringenin; Fluo: fluorescence; a.u.: arbitrary units CDS: coding DNA sequence; NA: not applicable, failed sequencing). Table S2. NUCLEOTIDE SEQUENCE OF THE CODON OPTIMIZED FJTAL, NEWLY SYNTHESIZED FOR THIS STUDY. Table S3. output of the artificial neural network (ANN) ensemble in the format described by Zhou et al. [30]. Pathway configurations are included in the Top x list if, after 1000 ANN train and predict iterations, the frequency of their occurrence in the predicted top x strains is higher than half the frequency of the most occurring strain in that top list ( f(top x) > 0.5*fmax(top x) ). Identical colors are used to indicate identical strains. (P_X: promoter variant of enzymatic step X; CDS_X: enzyme variant; freq: frequency (f)). Figure. S1 Characteristics of the naringenin-responsive biosensor (pSynSens1.100 [36]) in the conditions used in this study. (A) The responsive curve and fitted Hill function for a supplied naringenin concentration range of 0-100 mg/L and the corresponding Hill parameters. Also the operational range and Noise parameter are given, as determined with the method described by De Paepe et al. (2018) [36] and depicted in (B). (a: the basal normalized fluorescent signal (a.u., arbitrary units); M: the maximum normalized fluorescent signal (a.u.); n: Hill coefficient (cooperativity); K: Hill constant (transcription factor – ligand affinity, mg/L); error bars: standard errors for 5 biological replicates, n = 5). Figure. S2 Linlog transformation of the sigma B promoter library promoters as input for the created models. (A) Original data, displayed as sfGFP corrected mKate values [8] [file 12934_2022_1775_MOESM1_ESM.docx]

**Additional file 1**

**Additional file 1: Table S1:** Pathway architecture of the strains selected through the biosensor-driven combinatorial engineering process. The naringenin titer determined by UPLC analysis and the biosensor output is also given for each strain (n = 1). (TAL: Tyrosine ammonia-lyase; 4CL: 4-coumaroyl-CoA ligase; CHS: Chalcone synthase; CHI: Chalcone isomerase; Nar.: naringenin; Fluo: fluorescence; a.u.: arbitrary units CDS: coding DNA sequence; NA: not applicable, failed sequencing)

| **Strain ID** | **Part** | **TAL** |  | **4CL** |  | **CHS** |  | **CHI** |  | **Nar. (mg/L)** | **Fluo/OD_600_ (a.u.)** |
| --- | --- | --- | --- | --- | --- | --- | --- | --- | --- | --- | --- |
| **135** | Promoter | B5 |  | B3 |  | B10 |  | B1 |  | 27.0 ^a^ | 1737 |
|  | CDS |  | FjTAL |  | Pc4CL |  | GhCHS |  | MsCHI |  |  |
| **220** | Promoter | B4 |  | B1 |  | B10 |  | B6 |  | 16.3 | 672 |
|  | CDS |  | FjTAL |  | Pc4CL |  | GhCHS |  | MsCHI |  |  |
| **133** | Promoter | B4 |  | B1 |  | B6 |  | B7 |  | 16.1 | 1535 |
|  | CDS |  | FjTAL |  | Pc4CL |  | GhCHS |  | PhCHI |  |  |
| **195** | Promoter | B6 |  | B2 |  | B5 |  | B4 |  | 15.7 | 1524 |
|  | CDS |  | FjTAL |  | Pc4CL |  | GhCHS |  | MsCHI |  |  |
| **32** | Promoter | B4 |  | B1 |  | B9 |  | B8 |  | 15.6 | 945 |
|  | CDS |  | FjTAL |  | Pc4CL |  | GhCHS |  | PhCHI |  |  |
| **70** | Promoter | B3 |  | B3 |  | B6 |  | B3 |  | 14.2 | 1715 |
|  | CDS |  | FjTAL |  | Pc4CL |  | GhCHS |  | MsCHI |  |  |
| **232** | Promoter | B7 |  | B3 |  | B10 |  | B8 |  | 13.7 | 1979 |
|  | CDS |  | FjTAL |  | Pc4CL |  | NA |  | MsCHI |  |  |
| **104** | Promoter | B10 |  | B3 |  | B7 |  | B3 |  | 12.2 | 1495 |
|  | CDS |  | RgTAL |  | Pc4CL |  | GhCHS |  | MsCHI |  |  |
| **196** | Promoter | B4 |  | B2 |  | B10 |  | B6 |  | 11.9 | 935 |
|  | CDS |  | FjTAL |  | Pc4CL |  | GhCHS |  | MsCHI |  |  |
| **215** | Promoter | B6 |  | B3 |  | B6 |  | B5 |  | 11.4 | 4146 |
|  | CDS |  | FjTAL |  | Pc4CL |  | GhCHS |  | MsCHI |  |  |
| **203** | Promoter | B4 |  | B4 |  | B10 |  | B4 |  | 11.2 | 2124 |
|  | CDS |  | FjTAL |  | Pc4CL |  | GhCHS |  | MsCHI |  |  |

| **117** | Promoter | B5 |  | B4 |  | B10 |  | B2 |  | 11.2 | 2152 |
| --- | --- | --- | --- | --- | --- | --- | --- | --- | --- | --- | --- |
|  | CDS |  | FjTAL |  | Pc4CL |  | GhCHS |  | PhCHI |  |  |
| **231** | Promoter | B8 |  | B5 |  | B9 |  | B1 |  | 11.0 | 3860 |
|  | CDS |  | RgTAL |  | Pc4CL |  | GhCHS |  | MsCHI |  |  |
| **114** | Promoter | B5 |  | B5 |  | B4 |  | B3 |  | 10.7 | 1463 |
|  | CDS |  | RgTAL |  | Pc4CL |  | PhCHS |  | MsCHI |  |  |
| **17** | Promoter | B4 |  | B6 |  | B10 |  | B4 |  | 9.5 | 1248 |
|  | CDS |  | FjTAL |  | Pc4CL |  | GhCHS |  | MsCHI |  |  |
| **42** | Promoter | B6 |  | B2 |  | B6 |  | B2 |  | 9.2 | 347 |
|  | CDS |  | RgTAL |  | Pc4CL |  | GhCHS |  | MsCHI |  |  |
| **155** | Promoter | B4 |  | B6 |  | B10 |  | B2 |  | 8.1 | 4055 |
|  | CDS |  | RgTAL |  | Pc4CL |  | GhCHS |  | PhCHI |  |  |
| **148** | Promoter | B8 |  | B3 |  | B5 |  | B4 |  | 7.8 | 1847 |
|  | CDS |  | RgTAL |  | Pc4CL |  | GhCHS |  | MsCHI |  |  |
| **88** | Promoter | B5 |  | B6 |  | B10 |  | B7 |  | 7.6 | 1659 |
|  | CDS |  | FjTAL |  | Pc4CL |  | GhCHS |  | MsCHI |  |  |
| **43** | Promoter | B9 |  | B3 |  | B6 |  | B7 |  | 7.5 | 1140 |
|  | CDS |  | RgTAL |  | Pc4CL |  | GhCHS |  | PhCHI |  |  |
| **110** | Promoter | B4 |  | B8 |  | B8 |  | B6 |  | 7.1 | 507 |
|  | CDS |  | RgTAL |  | Pc4CL |  | PhCHS |  | MsCHI |  |  |
| **84** | Promoter | B10 |  | B3 |  | B4 |  | B4 |  | 7.1 | 2090 |
|  | CDS |  | RgTAL |  | Pc4CL |  | GhCHS |  | PhCHI |  |  |
| **193** | Promoter | B4 |  | B5 |  | B5 |  | B2 |  | 6.7 | 1380 |
|  | CDS |  | RgTAL |  | Pc4CL |  | GhCHS |  | MsCHI |  |  |
| **63** | Promoter | B5 |  | B8 |  | B9 |  | B6 |  | 6.5 | 3494 |
|  | CDS |  | RgTAL |  | Pc4CL |  | GhCHS |  | PhCHI |  |  |
| **81** | Promoter | B5 |  | B6 |  | B7 |  | B2 |  | 6.4 | 2393 |
|  | CDS |  | RgTAL |  | Pc4CL |  | GhCHS |  | MsCHI |  |  |

| **Strain ID** | **Part** | **TAL** |  | **4CL** |  | **CHS** |  | **CHI** |  | **Nar. (mg/L)** | **Fluo/OD_600_ (a.u.)** |
| --- | --- | --- | --- | --- | --- | --- | --- | --- | --- | --- | --- |
| **93** | Promoter | B4 |  | B6 |  | B10 |  | B4 |  | 5.8 | 2542 |
|  | CDS |  | RgTAL |  | Pc4CL |  | GhCHS |  | MsCHI |  |  |
| **22** | Promoter | B4 |  | B1 |  | B4 |  | B3 |  | 5.4 | 178 |
|  | CDS |  | RgTAL |  | Pc4CL |  | GhCHS |  | MsCHI |  |  |
| **80** | Promoter | B3 |  | B6 |  | B5 |  | B2 |  | 5.4 | 1261 |
|  | CDS |  | FjTAL |  | Pc4CL |  | GhCHS |  | PhCHI |  |  |
| **125** | Promoter | B2 |  | B1 |  | B2 |  | B7 |  | 5.3 | 5479 |
|  | CDS |  | FjTAL |  | Pc4CL |  | GhCHS |  | PhCHI |  |  |
| **30** | Promoter | B3 |  | B3 |  | B9 |  | B5 |  | 4.5 | 221 |
|  | CDS |  | RgTAL |  | Pc4CL |  | GhCHS |  | MsCHI |  |  |
| **66** | Promoter | B3 |  | B6 |  | B9 |  | B7 |  | 4.1 | 1005 |
|  | CDS |  | RgTAL |  | Pc4CL |  | GhCHS |  | PhCHI |  |  |
| **77** | Promoter | B3 |  | B5 |  | B4 |  | B4 |  | 3.8 | 825 |
|  | CDS |  | RgTAL |  | Pc4CL |  | GhCHS |  | MsCHI |  |  |
| **127** | Promoter | B3 |  | B1 |  | B7 |  | B4 |  | 3.4 | 573 |
|  | CDS |  | RgTAL |  | Pc4CL |  | PhCHS |  | MsCHI |  |  |
| **74** | Promoter | B3 |  | B4 |  | B7 |  | B10 |  | 3.2 | 1042 |
|  | CDS |  | RgTAL |  | At4CL |  | GhCHS |  | PhCHI |  |  |
| **60** | Promoter | B1 |  | B7 |  | B4 |  | B5 |  | 1.4 | 72 |
|  | CDS |  | RgTAL |  | Pc4CL |  | GhCHS |  | PhCHI |  |  |

^a^ Suspected biological outlier based on further characterization.

**Additional file 1: Table S2:** Nucleotide sequence of the codon optimized FjTAL, newly synthesized for this study.

| ***FjTAL*:** *Flavobacterium johnsoniae* tyrosine ammonia-lyase |
| --- |
| 5’‑ atgAACACCATCAACGAATATCTGAGCCTGGAAGAATTTGAAGCCATTATCTTTGGCAATCAGAAAGTGACCATTAGTGATGTTGTTGTGAATCGCGTTAACGAGAGCTTTAACTTTCTGAAAGAATTTAGCGGCAACAAAGTGATCTATGGTGTGAATACCGGTTTTGGTCCGATGGCACAGTATCGTATTAAAGAAAGCGATCAGATTCAGCTGCAGTATAATCTGATTCGTAGCCATAGCAGCGGCACCGGTAAACCGCTGAGTCCGGTTTGTGCAAAAGCAGCAATTCTGGCACGTCTGAATACCCTGAGTCTGGGTAATAGCGGTGTTCATCCGAGCGTTATTAATCTGATGAGCGAACTGATCAACAAAGATATCACACCGCTGATTTTTGAACATGGTGGTGTTGGTGCAAGCGGTGATCTGGTTCAGCTGAGCCATCTGGCACTGGTTCTGATTGGTGAAGGTGAAGTTTTCTATAAAGGTGAACGTCGTCCGACACCGGAAGTTTTTGAAATTGAAGGTCTGAAACCGATCCAGGTGGAAATTCGCGAAGGTCTGGCCCTGATTAATGGCACCAGCGTTATGACCGGTATTGGTGTTGTTAATGTGTACCATGCAAAAAAACTGCTGGATTGGAGCCTGAAAAGCAGCTGTGCAATTAATGAACTGGTTCAGGCATATGATGATCACTTTAGCGCAGAACTGAATCAGACCAAACGTCATAAAGGTCAGCAAGAAATTGCACTGAAAATGCGTCAGAATCTGAGCGATAGCACCCTGATTCGCAAACGTGAAGATCATCTGTATAGCGGTGAAAACACCGAAGAAATCTTCAAAGAAAAAGTGCAAGAGTATTATAGCCTGCGTTGTGTTCCGCAGATTCTGGGTCCGGTTCTGGAAACCATTAACAATGTTGCAAGCATTCTGGAAGATGAATTTAACAGCGCAAACGATAACCCGATCATCGATGTTAAAAACCAGCATGTTTATCACGGTGGCAATTTTCATGGTGATTATATCAGCCTGGAAATGGATAAACTGAAAATCGTGATTACCAAACTGACCATGCTGGCAGAACGTCAGCTGAATTATCTGCTGAATAGCAAAATTAACGAACTGCTGCCTCCGTTTGTTAATCTGGGCACCCTGGGTTTTAACTTTGGTATGCAGGGTGTTCAGTTTACCGCAACCAGCACCACCGCAGAAAGCCAGATGCTGAGCAATCCGATGTATGTTCATAGCATTCCGAACAATAATGATAACCAGGATATTGTTAGCATGGGCACCAATAGCGCAGTTATTACCAGCAAAGTTATCGAAAATGCCTTTGAAGTTCTGGCCATTGAAATGATTACCATTGTTCAGGCGATTGATTATCTGGGCCAGAAAGATAAAATCAGCAGCGTTAGCAAAAAATGGTATGATGAAATCCGCAACATCATCCCGACCTTTAAAGAAGATCAGGTGATGTATCCGTTCGTGCAGAAAGTAAAAGACCACCTGATTAACAATtaa ‑3’ |

**Additional file 1: Table S3:** Output of the artificial neural network (ANN) ensemble in the format described by Zhou *et al.* (30). Pathway configurations are included in the Top *x* list if, after 1000 ANN train and predict iterations, the frequency of their occurrence in the predicted top *x* strains is higher than half the frequency of the most occurring strain in that top list ( f(top *x*) > 0.5*f_max_(top *x*) ). Identical colors are used to indicate identical strains. (P_*X*: promoter variant of enzymatic step *X*; CDS_*X*: enzyme variant; freq: frequency (f))

| **Top 1 Producers** | **P_TAL** | **P_4CL** | **P_CHS** | **P_CHI** | **CDS_TAL** | **CDS_CHI** | **Top1_freq** | **Top5_freq** | **Top10_freq** |
| --- | --- | --- | --- | --- | --- | --- | --- | --- | --- |
|  | B10 | B1 | B10 | B1 | FjTAL | MsCHI | 0.393 | 0.091 | 0.0502 |
|  |  |  |  |  |  |  |  |  |  |
| **Top 5 Producers** | **P_TAL** | **P_4CL** | **P_CHS** | **P_CHI** | **CDS_TAL** | **CDS_CHI** | **Top1_freq** | **Top5_freq** | **Top10_freq** |
|  | B10 | B1 | B10 | B1 | FjTAL | MsCHI | 0.393 | 0.091 | 0.0502 |
|  | B9 | B1 | B10 | B1 | FjTAL | MsCHI | 0.004 | 0.075 | 0.0462 |
|  | B10 | B1 | B9 | B1 | FjTAL | MsCHI | 0.007 | 0.063 | 0.0421 |
|  | B8 | B1 | B10 | B1 | FjTAL | MsCHI | 0.005 | 0.0538 | 0.0415 |
|  |  |  |  |  |  |  |  |  |  |
| **Top 10 Producers** | **P_TAL** | **P_4CL** | **P_CHS** | **P_CHI** | **CDS_TAL** | **CDS_CHI** | **Top1_freq** | **Top5_freq** | **Top10_freq** |
|  | B10 | B1 | B10 | B1 | FjTAL | MsCHI | 0.393 | 0.091 | 0.0502 |
|  | B9 | B1 | B10 | B1 | FjTAL | MsCHI | 0.004 | 0.075 | 0.0462 |
|  | B10 | B1 | B9 | B1 | FjTAL | MsCHI | 0.007 | 0.063 | 0.0421 |
|  | B8 | B1 | B10 | B1 | FjTAL | MsCHI | 0.005 | 0.0538 | 0.0415 |
|  | B10 | B1 | B8 | B1 | FjTAL | MsCHI | 0 | 0.0452 | 0.0341 |
|  | B9 | B1 | B9 | B1 | FjTAL | MsCHI | 0 | 0.0064 | 0.0309 |


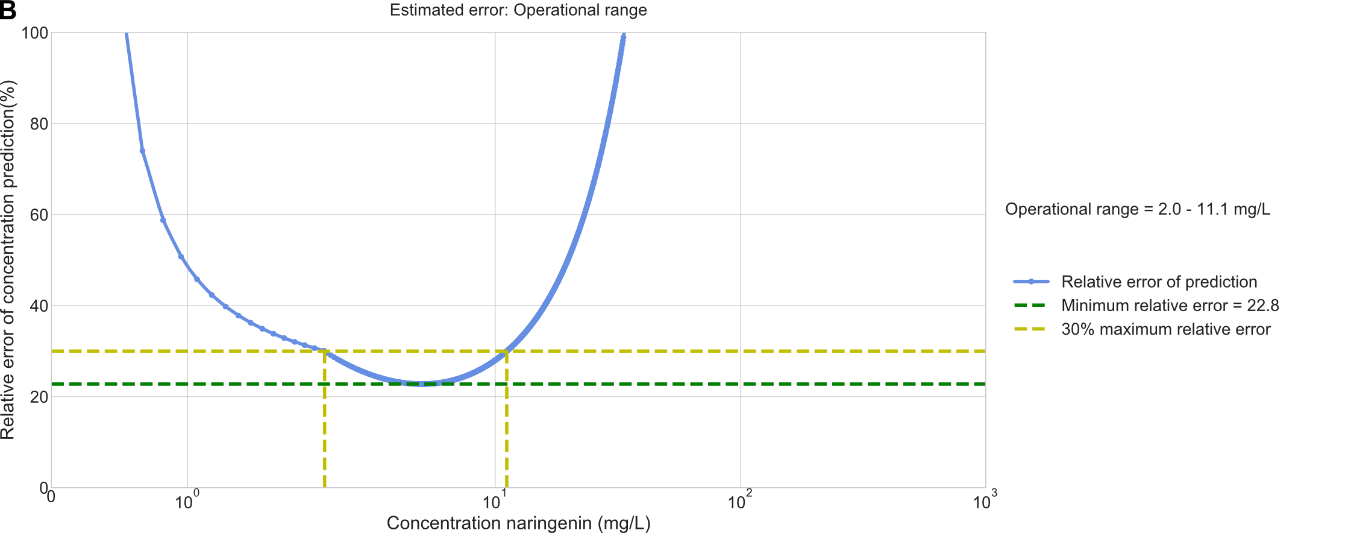


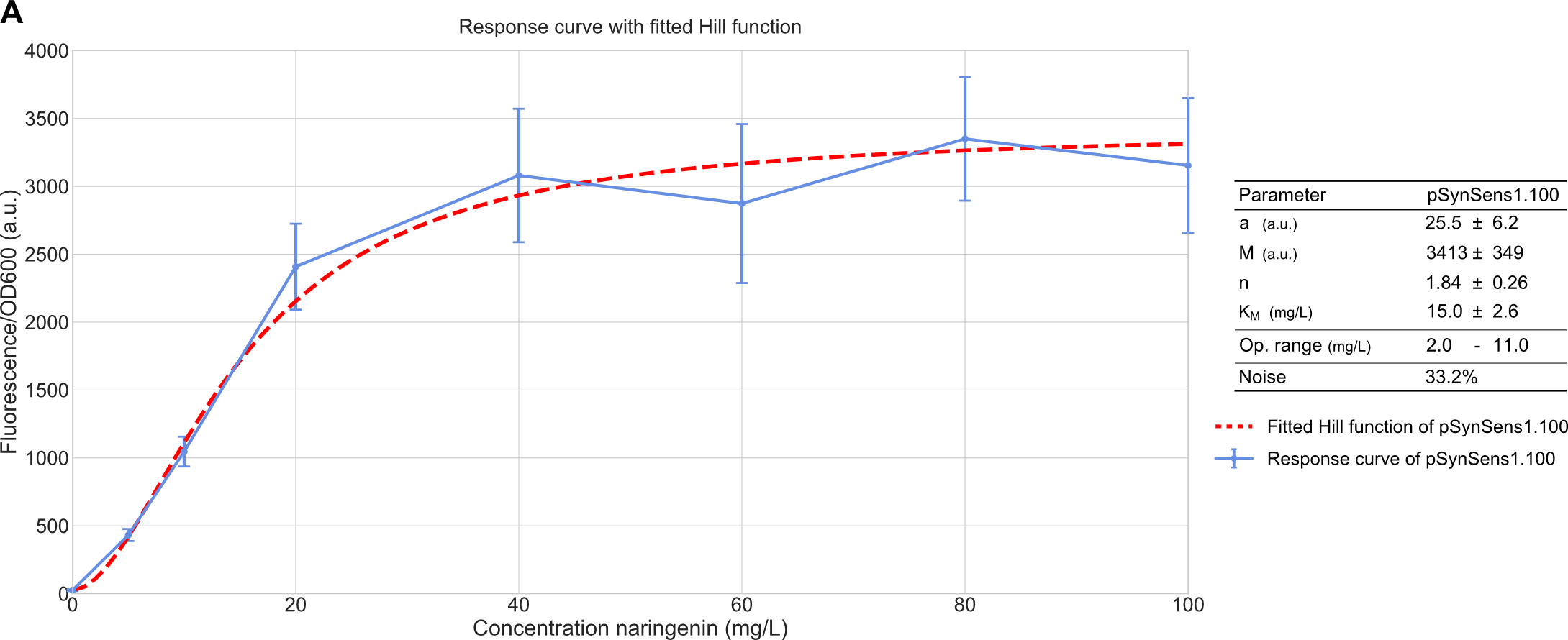


**Additional file 1: Figure S1:** Characteristics of the naringenin-responsive biosensor (pSynSens1.100 (36)) in the conditions used in this study. (**A**) The responsive curve and fitted Hill function for a supplied naringenin concentration range of 0-100 mg/L and the corresponding Hill parameters. Also the operational range and *Noise* parameter are given, as determined with the method described by De Paepe *et al.* (2018) (36) and depicted in (**B**). (*a*: the basal normalized fluorescent signal (a.u., arbitrary units); *M*: the maximum normalized fluorescent signal (a.u.); *n*: Hill coefficient (cooperativity); *K*: Hill constant (transcription factor – ligand affinity, mg/L); error bars: standard errors for 5 biological replicates, n = 5).


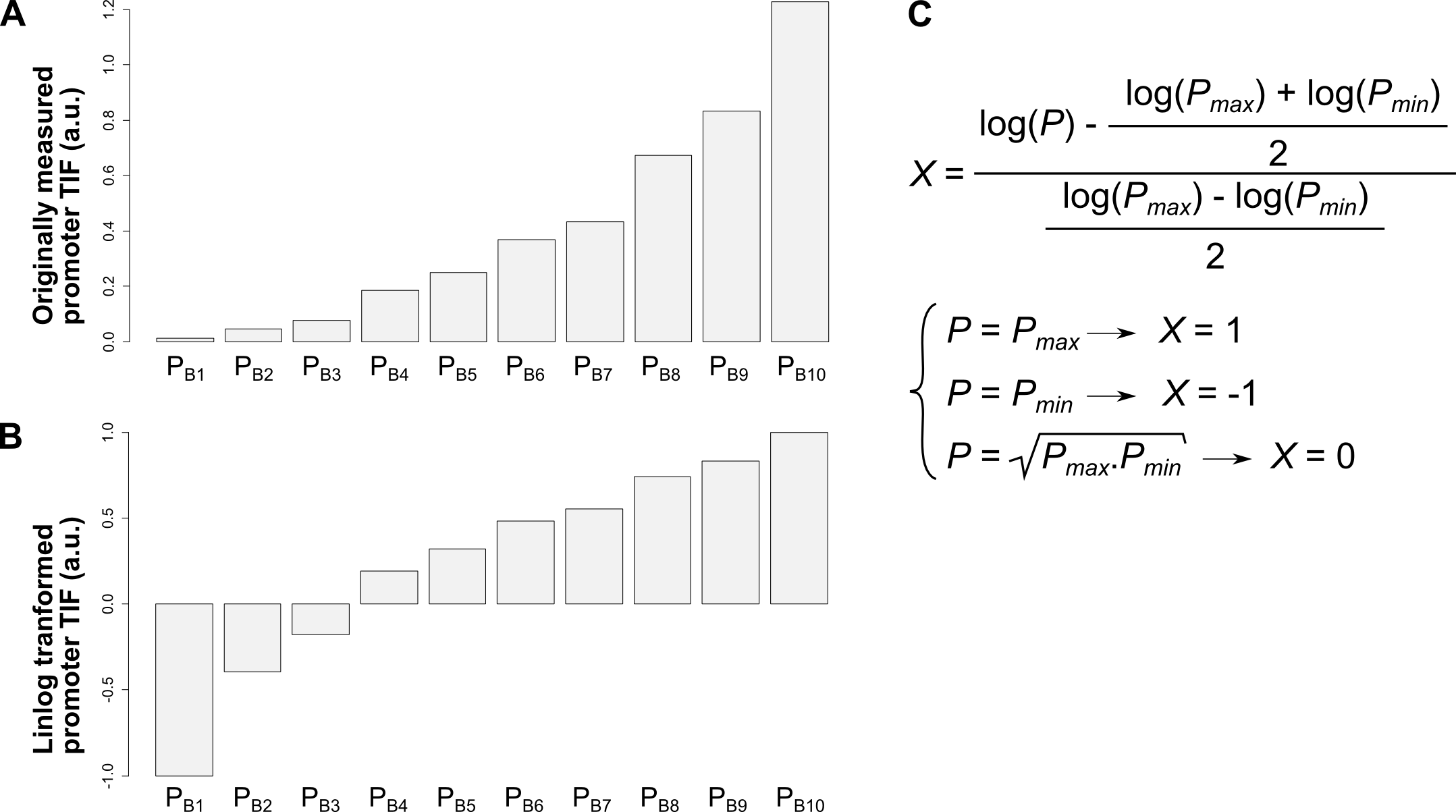


**Additional file 1: Figure S2:** Linlog transformation of the sigma B promoter library promoters as input for the created models. (**A**) Original data, displayed as sfGFP corrected mKate values (8). (B) Linlog transformed promoters. (**C**) Used linlog transformation and properties of the transformed data. (TIF: transcription initiation frequency; *P* = untransformed promoter TIF, *X* = linlog transformed promoter TIF).

Correlation matrix

P_TAL P_4CL P_CHS P_CHI Titer

P_TAL 1.00 -0.06 0.18 -0.21 0.39

P_4CL -0.06 1.00 0.25 -0.19 -0.35

P_CHS 0.18 0.25 1.00 0.00 0.35

P_CHI -0.21 -0.19 0.00 1.00 -0.26

Titer 0.39 -0.35 0.35 -0.26 1.00

Sample Size

[1] 35

Probability values (Entries above the diagonal are adjusted for multiple tests.)

P_TAL P_4CL P_CHS P_CHI Titer

P_TAL 0.00 1.00 1.00 1.00 0.22

P_4CL 0.71 0.00 0.94 1.00 0.34

P_CHS 0.29 0.15 0.00 1.00 0.34

P_CHI 0.23 0.28 0.99 0.00 0.94

Titer 0.02 0.04 0.04 0.13 0.00

**Additional file 1: Figure S3:** Correlation (Pearson, ρ) between the continuous variables (promoter transcription initiation frequency and titer) in the dataset, shown as the generated output of the corr.test() function of the R psych package (71). The top matrix shows the correlation between the variables with -1 and 1 indicating a perfect (inverse) correlation and 0, no correlation. The bottom matrix shows the corresponding probability values (Null hypothesis = ‘H_0_: 2 variables are not correlated (ρ = 0)’). The Holm-Bonferroni method is used to adjust for multiple testing. (Rg: *Rhodotorula glutinis*; Fj: *Flavobacterium johnsoniae*; Pc: *Petroselinum crispum*; At: *Arabidopsis thaliana*; Ph: *Petunia hybrida*; Gh: *Gerbera hybrida*; Ms: *Medicago sativa*; TAL: Tyrosine ammonia-lyase; 4CL: 4-coumaroyl-CoA ligase; CHS: Chalcone synthase; CHI: Chalcone isomerase).

**Formula:** Titer ~ P_TAL + P_4CL + P_CHS + P_CHI +
 CDS_TAL + CDS_CHI +
 I(P_4CL^2) +
 P_CHI:CDS_CHI +
 P_TAL:P_CHS + P_4CL:P_CHS + P_4CL:P_CHI +
 P_CHS:P_CHI

Residuals:

Min 1Q Median 3Q Max

-2.0952 -0.8163 -0.3224 0.9515 2.8211

Coefficients:

Estimate Std. Error t value Pr(>|t|)

(Intercept) 9.0639 1.1071 8.187 2.66e-07 ***

P_TAL 6.8998 2.0309 3.397 0.003427 **

P_4CL 2.9113 1.9463 1.496 0.153043

P_CHS 5.7718 1.3177 4.380 0.000408 ***

P_CHI 4.9908 3.2107 1.554 0.138505

CDS_TALRgTAL -6.3582 0.8596 -7.396 1.04e-06 ***

CDS_CHIPhCHI -1.8599 0.9482 -1.961 0.066421 .

I(P_4CL^2) 3.2148 1.9957 1.611 0.125617

P_CHI:CDS_CHIPhCHI 6.2274 1.8637 3.341 0.003868 **

P_TAL:P_CHS -8.0584 4.2907 -1.878 0.077632 .

P_4CL:P_CHS -8.4995 2.0082 -4.232 0.000561 ***

P_4CL:P_CHI 3.8063 1.8680 2.038 0.057456 .

P_CHS:P_CHI -15.1544 3.9426 -3.844 0.001301 **

---

Significance codes:

0 ‘***’ 0.001 ‘**’ 0.01 ‘*’ 0.05 ‘.’ 0.1 ‘ ’ 1

Residual standard error: 1.773 on 17 degrees of freedom

Multiple R-squared: 0.9316, Adjusted R-squared: 0.8833

F-statistic: 19.29 on 12 and 17 DF, p-value: 1.479e-07

**Additional file 1: Figure S4:** Ordinary least squares regression output from the lm() function in R. The input formula is obtained by a limitted sequential removal of terms holding the highest p-value, starting from the full quadratic regression model. (Q: quadrant; P_*X*: promoter transcription initiation frequency for expression of enzyme *X*; CDS_X: coding sequence variant of enzyme *X*; P_*X***:**P_*X*: interaction term; I(P_*X*^2): quadratic term; Rg: *Rhodotorula glutinis*; Fj: *Flavobacterium johnsoniae*; Pc: *Petroselinum crispum*; At: *Arabidopsis thaliana*; Ph: *Petunia hybrida*; Gh: *Gerbera hybrida*; Ms: *Medicago sativa*; TAL: Tyrosine ammonia-lyase; 4CL: 4-coumaroyl-CoA ligase; CHS: Chalcone synthase; CHI: Chalcone isomerase).


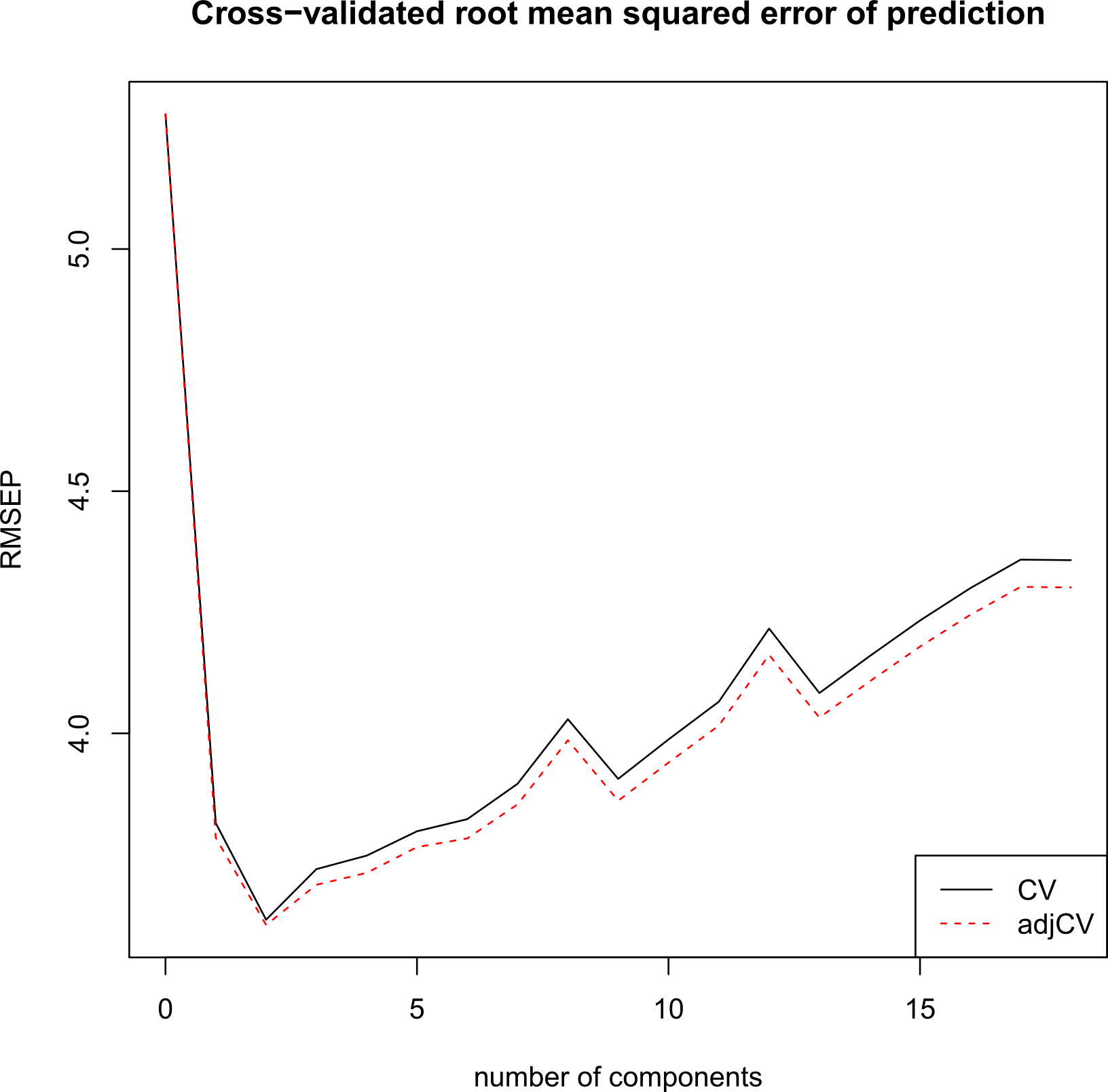


**Additional file 1: Figure S5:** Cross-validated (CV) root mean squared error of prediction (RMSEP) curve. A model only including the first two components (*i.e.* latent variables, LV) shows the lowest prediction error. A model with two LV predicts 78.92% of the product titer by using 38.82% of the predictors’ variance. (adjCV: adjusted CV).


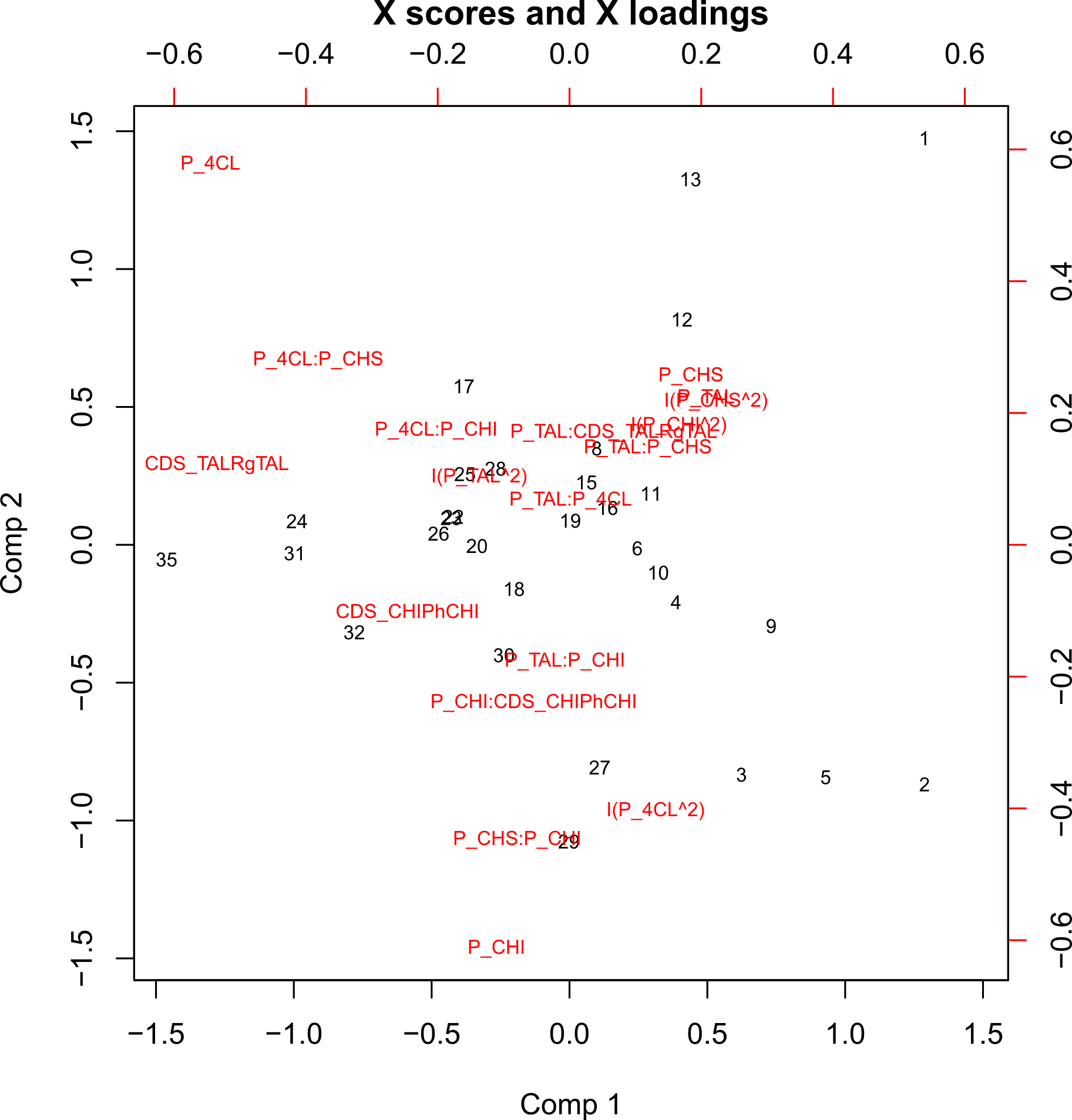


**Additional file 1: Figure S6:** Biplot of the first two components of the partial least squares (PLS) regression model. (P_*X*: promoter transcription initiation frequency (TIF) of enzymatic step *X*; CDS_*X*: enzyme variant; I(P_*X*^2): quadratic term of promoter transcription initiation frequency; ‘P_*X*:CDS_*X*’ and ‘P_*X*: P_*Y*’: promoter TIF interaction terms with enzyme variants or between the promoter TIFs of two different enzymatic pathway reaction steps; TAL: Tyrosine ammonia-lyase; 4CL: 4-coumaroyl-CoA ligase; CHS: Chalcone synthase; CHI: Chalcone isomerase).


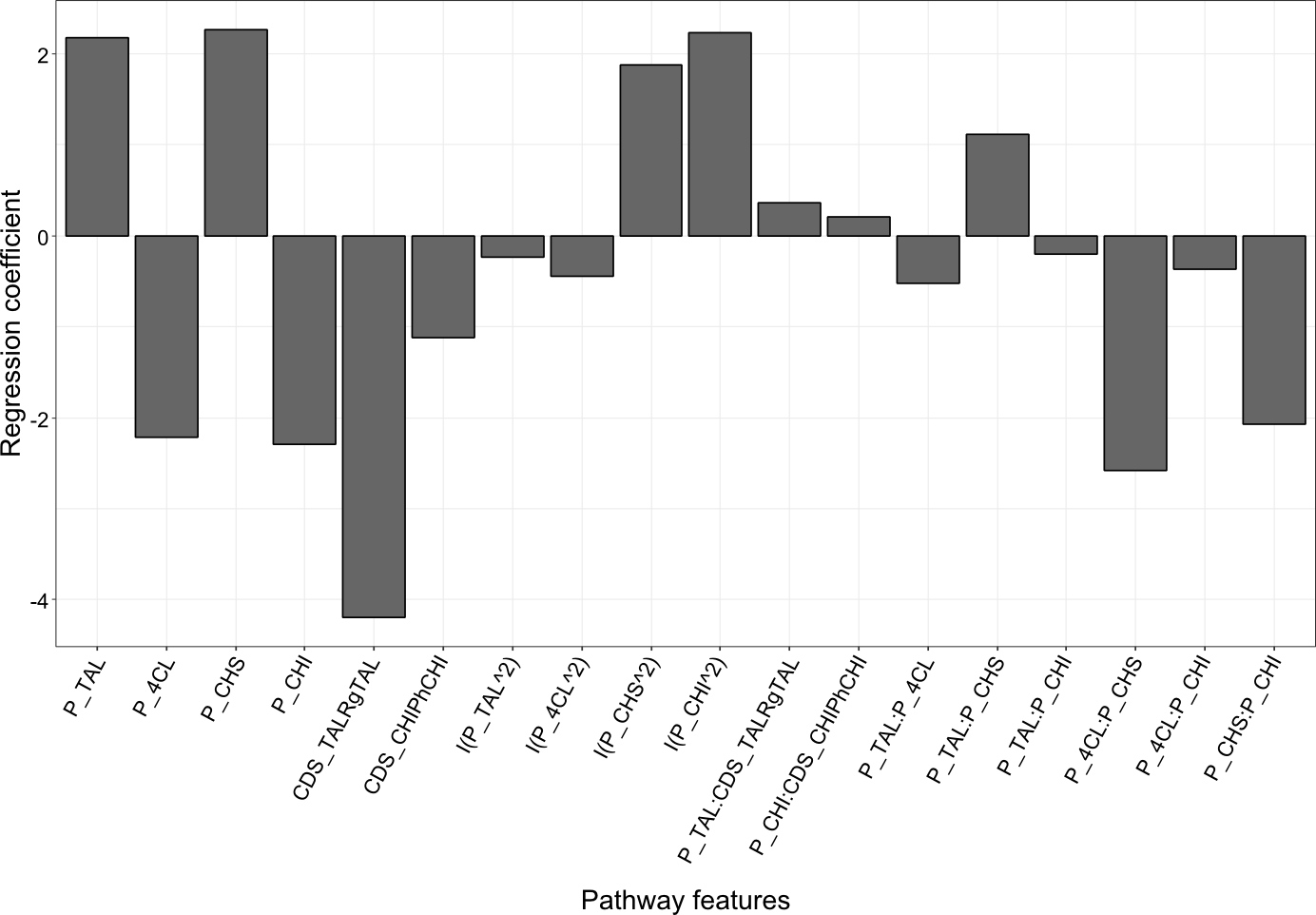


**Additional file 1: Figure S7:** The estimated partial least squares (PLS) regression coefficients of all pathway features, including quadratic and interaction terms. (P_*X*: promoter transcription initiation frequency (TIF) of enzymatic step *X*; CDS_*X*: enzyme variant; I(P_*X*^2): quadratic term of promoter TIF; ‘P_*X*:CDS_*X*’ and ‘P_*X*: P_*Y*’: promoter TIF interaction terms with enzyme variants or between the promoter TIFs of two different enzymatic pathway reaction steps; TAL: Tyrosine ammonia-lyase; 4CL: 4-coumaroyl-CoA ligase; CHS: Chalcone synthase; CHI: Chalcone isomerase).


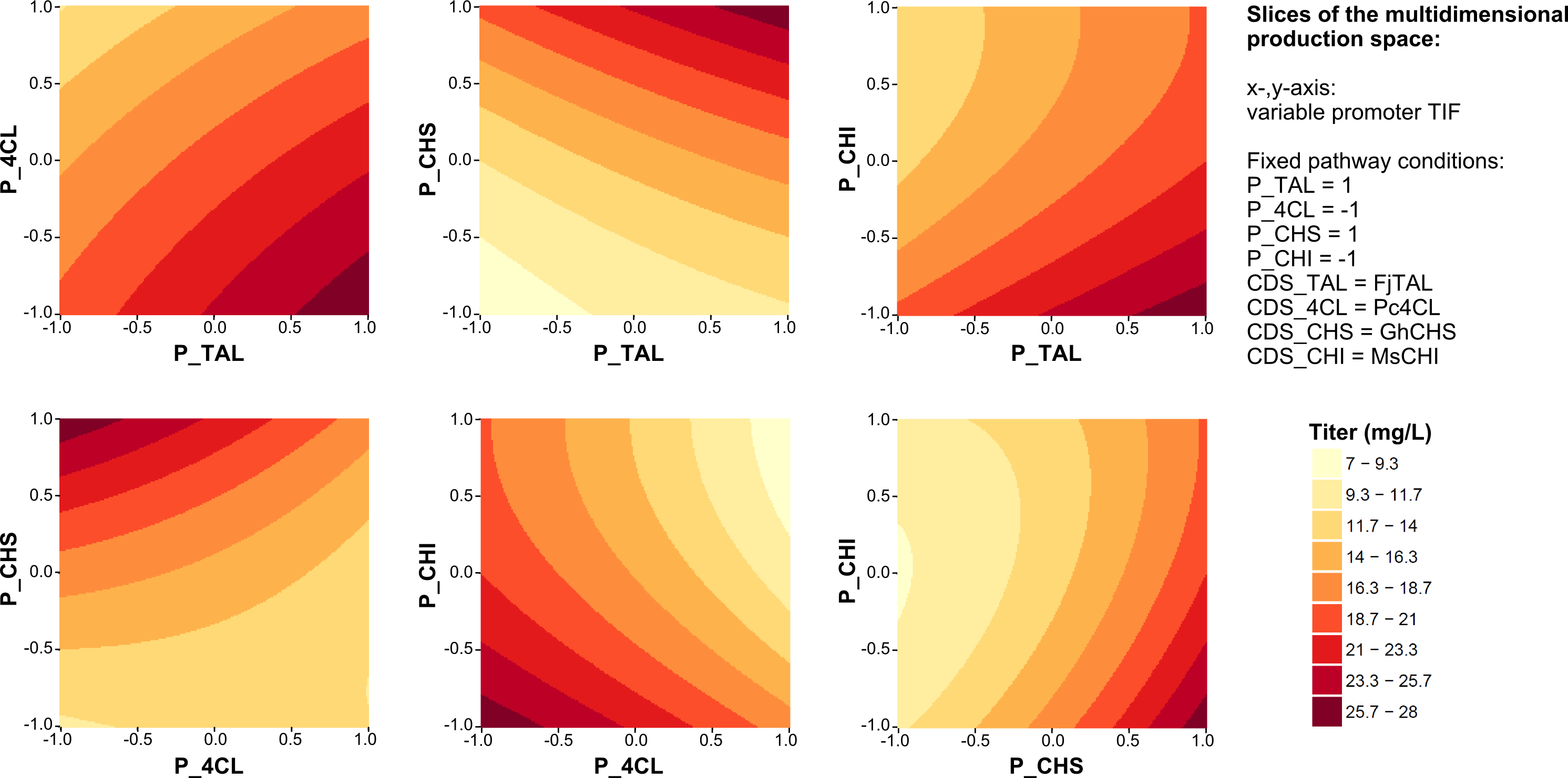


**Additional file 1: Figure S8:** Cross sections of the multidimensional production landscape, predicted by the partial least squares (PLS) model. For each cross section, two pathway features (promoter transcription initiation frequencies, TIF) are varied while the remaining part of the pathway configuration is fixed. The fixed input values, other than the two variables depicted on the x- and y-axes, are set according to the predicted optimal producer (see legend, P_*X*: promoter TIF of enzymatic step *X*; CDS_*X*: enzyme variant; TAL: Tyrosine ammonia-lyase; 4CL: 4-coumaroyl-CoA ligase; CHS: Chalcone synthase; CHI: Chalcone isomerase).


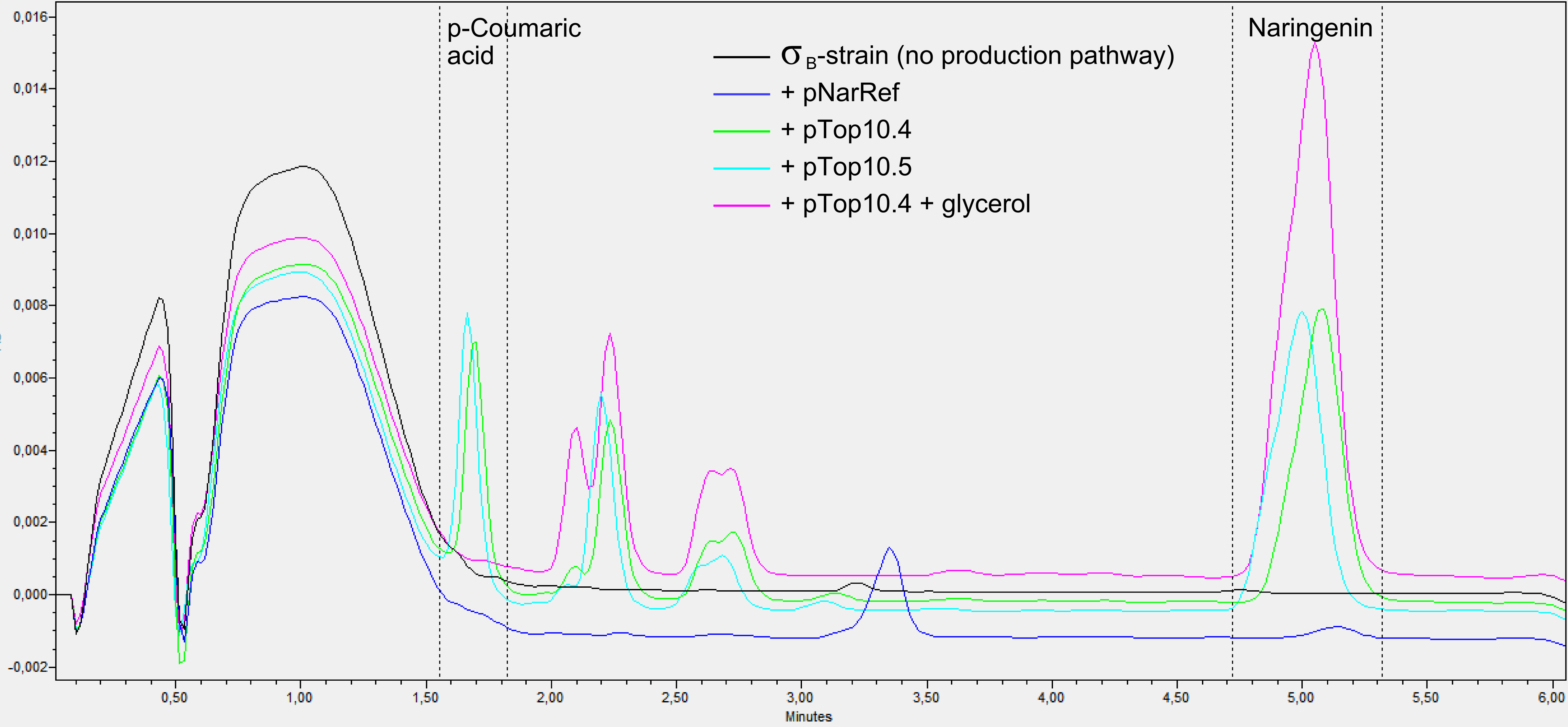


**Additional file 1: Figure S9:** The UPLC-UV chromatogram of one of the ethyl acetate-extracted biological replications of NarRef, Top10.4, Top10.5 and Top10.4 grown on the glycerol supplied medium. The Top10.5 + glycerol profile is similar to the Top10.4 + glycerol profile and the depicted profiles are also representative for the other biological replications, but are left out for visual clarity. As a reference, also the chromatographic profile of the strain bearing only heterologous sigma factor (σ) B in the genome but no plasmid is included.


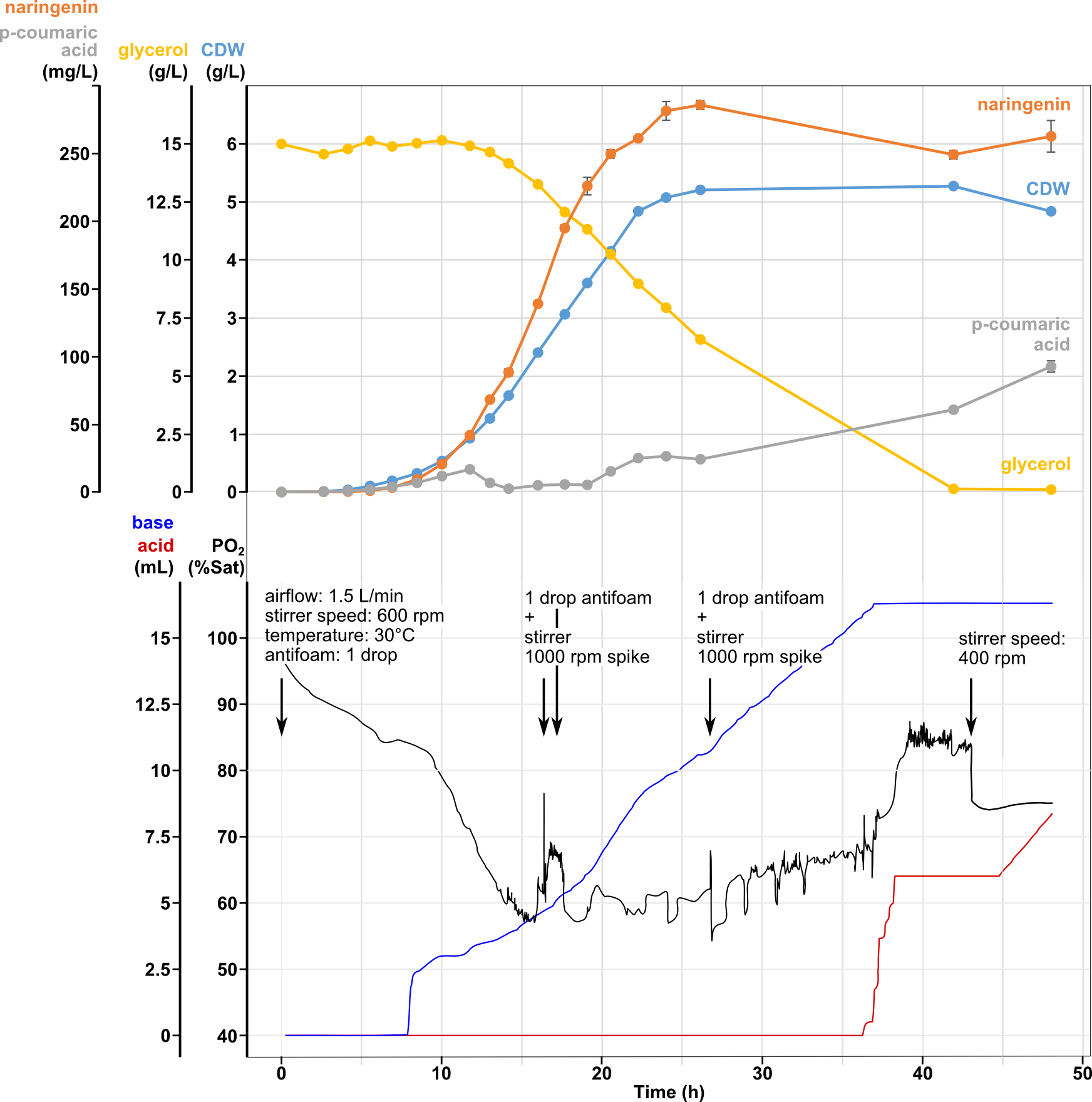


**Additional file 1: Figure S10:** Batch fermentation with production strain Top10.4. In the upper part the production profiles of cell dry weight (CDW), naringenin and *p*-coumaric acid, and the substrate usage of glycerol are given. In the lower part, the process parameter profiles for base and acid addition and dissolved oxygen (PO_2_) are given, together with events of process parameter(-change) indications for airflow, stirrer speed, temperature and antifoam addition. Stirrer speed spikes were used to break accumulated foam.


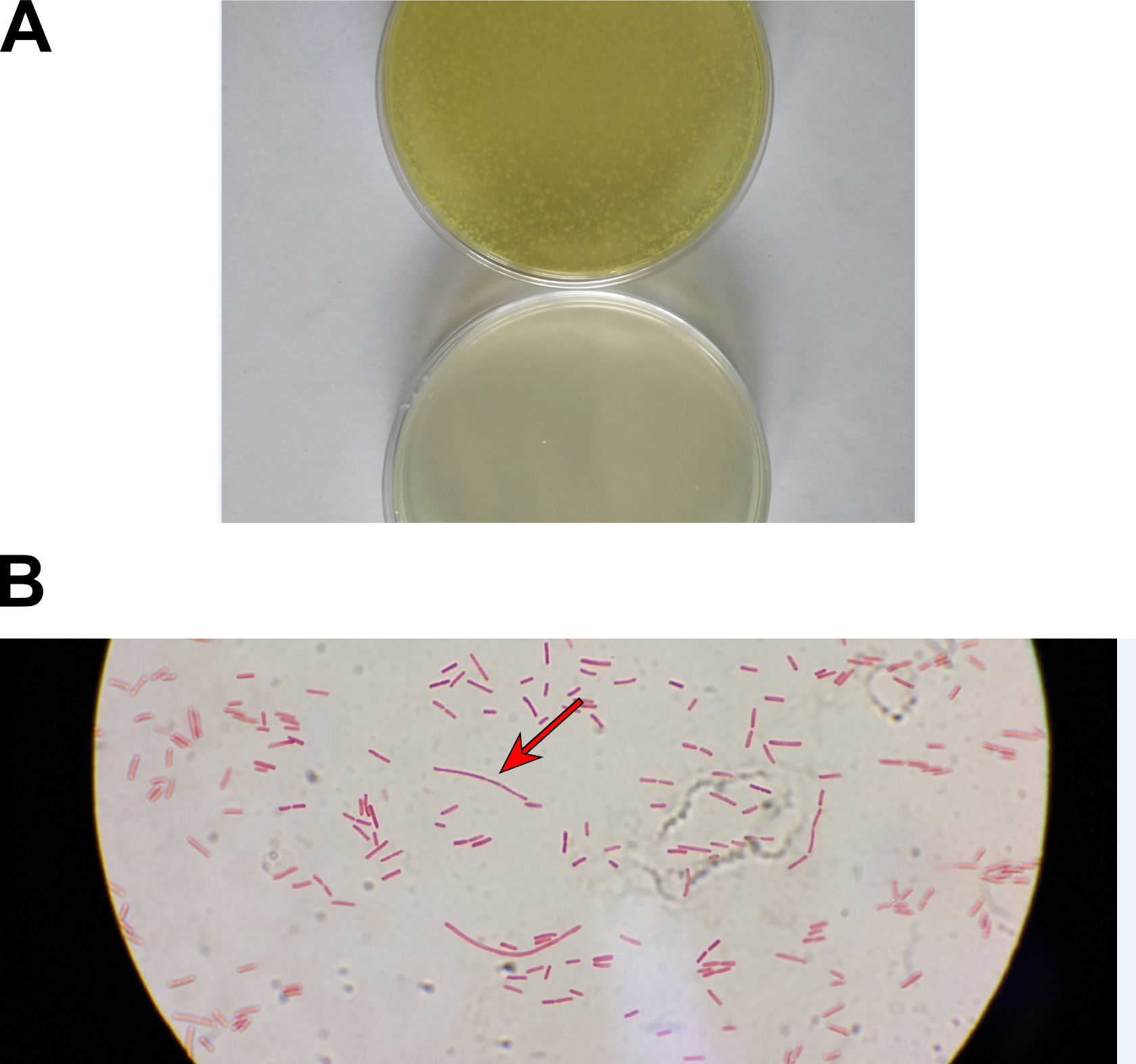


**Additional file 1: Figure S11:** (**A**) Plating of the first sample taken of the batch fermentation with production strain Top10.4, compared to an empty LB-agar plate. The yellow coloration is found to indicate product formation, most likely coming from intermediate metabolite naringenin chalcone. (**B**) Gram-stained sample of the performed batch fermentation with strain Top10.4. Filamentation of the production organism is an indication for stress (52).

LOCUS **pNar-Top10.4** 9884 bp ds-DNA circular

DEFINITION .

FEATURES Location/Qualifiers

misc_feature 1..200

/label="L3"

misc_feature 203..247

/label="insulator region, Davis et al. (2011)"

misc_feature 212

/label="mut A -> G"

promoter 248..306

/label="SigmaB8 - Promoter"

-35_signal 273..278

/label="-35 box"

-10_signal 291..296

/label="-10 box"

RBS 307..394

/label="BCD19"

CDS 392..1912

/label="FjTAL"

terminator 1948..2037

/label="rrnD1_T1"

misc_feature 2040..2069

/label="L4'"

misc_feature 2100..2299

/label="L4"

misc_feature 2302..2346

/label="insulator region, Davis et al. (2011)"

misc_feature 2311

/label="mut A -> G"

promoter 2347..2405

/label="SigmaB1 - Promoter"

-35_signal 2372..2377

/label="-35 box"

-10_signal 2390..2395

/label="-10 box"

RBS 2406..2493

/label="BCD19"

CDS 2491..4125

/label="Pc4CL"

terminator 4158..4248

/label="rrnB_T1"

misc_feature 4249..4284

/label="L5'"

misc_feature 4315..4514

/label="L5"

misc_feature 4517..4561

/label="insulator region, Davis et al. (2011)"

misc_feature 4526

/label="mut A -> G"

promoter 4562..4622

/label="SigmaB10 - Promoter"

-35_signal 4587..4592

/label="-35 box"

-10_signal 4607..4612

/label="-10 box"

RBS 4623..4710

/label="BCD19"

CDS 4708..5904

/label="GhCHS"

terminator 5937..6023

/label="trp_att_L126"

misc_feature 6024..6068

/label="L6'"

misc_feature 6099..6298

/label="L6"

misc_feature 6301..6345

/label="insulator region, Davis et al. (2011)"

misc_feature 6310

/label="mut A -> G"

promoter 6346..6404

/label="SigmaB1 - Promoter"

-35_signal 6371..6376

/label="-35 box"

-10_signal 6389..6394

/label="-10 box"

RBS 6405..6492

/label="BCD19"

CDS 6490..7158

/label="MsCHI"

terminator 7191..7278

/label="T7_Te"

misc_feature 7279..7299

/label="L7'"

CDS 7515..8309

/label="Tn5"

misc_feature complement(9047..9599)

/label="RNAII"

rep_origin complement(9047..9635)

/label="pMB1"

misc_feature 9489..9596

/label="RNAI"

ORIGIN

1 GTGCAAAGTT TCATAAACTA CTTCCTTTCG CATGATACAT AACATTACGT TTACGGGAGT

61 GCAGTGTGAC CCACTAACCA GCGGAAGGTT AGCTGACCAC CTTCACGTCG TGCTATCGAA

121 ATCCTCTCAG TTGGCACACT TTACGACAGA AACTACCAGG CCAGGAGTCC TTCTCGATGC

181 TCTGGCTTCT CCGGAGATTC CCTTCTAGAG CGCAGCTAAC ACCACGTCGT CCCTATCTGC

241 TGCCCTATGT TTAAAAAAAT GTCGGAGAAC GTGTTTATTC TGGGAAAATC GGGTATGTAA

301 CTTGTAGGGC CCAAGTTCAC TTAAAAAGGA GATCAACAAT GAAAGCAATT TTCGTACTGA

361 AACATCTTAA TCATGCTATG GAGGTTTTCT AATGAACACC ATCAACGAAT ATCTGAGCCT

421 GGAAGAATTT GAAGCCATTA TCTTTGGCAA TCAGAAAGTG ACCATTAGTG ATGTTGTTGT

481 GAATCGCGTT AACGAGAGCT TTAACTTTCT GAAAGAATTT AGCGGCAACA AAGTGATCTA

541 TGGTGTGAAT ACCGGTTTTG GTCCGATGGC ACAGTATCGT ATTAAAGAAA GCGATCAGAT

601 TCAGCTGCAG TATAATCTGA TTCGTAGCCA TAGCAGCGGC ACCGGTAAAC CGCTGAGTCC

661 GGTTTGTGCA AAAGCAGCAA TTCTGGCACG TCTGAATACC CTGAGTCTGG GTAATAGCGG

721 TGTTCATCCG AGCGTTATTA ATCTGATGAG CGAACTGATC AACAAAGATA TCACACCGCT

781 GATTTTTGAA CATGGTGGTG TTGGTGCAAG CGGTGATCTG GTTCAGCTGA GCCATCTGGC

841 ACTGGTTCTG ATTGGTGAAG GTGAAGTTTT CTATAAAGGT GAACGTCGTC CGACACCGGA

901 AGTTTTTGAA ATTGAAGGTC TGAAACCGAT CCAGGTGGAA ATTCGCGAAG GTCTGGCCCT

961 GATTAATGGC ACCAGCGTTA TGACCGGTAT TGGTGTTGTT AATGTGTACC ATGCAAAAAA

1021 ACTGCTGGAT TGGAGCCTGA AAAGCAGCTG TGCAATTAAT GAACTGGTTC AGGCATATGA

1081 TGATCACTTT AGCGCAGAAC TGAATCAGAC CAAACGTCAT AAAGGTCAGC AAGAAATTGC

1141 ACTGAAAATG CGTCAGAATC TGAGCGATAG CACCCTGATT CGCAAACGTG AAGATCATCT

1201 GTATAGCGGT GAAAACACCG AAGAAATCTT CAAAGAAAAA GTGCAAGAGT ATTATAGCCT

1261 GCGTTGTGTT CCGCAGATTC TGGGTCCGGT TCTGGAAACC ATTAACAATG TTGCAAGCAT

1321 TCTGGAAGAT GAATTTAACA GCGCAAACGA TAACCCGATC ATCGATGTTA AAAACCAGCA

1381 TGTTTATCAC GGTGGCAATT TTCATGGTGA TTATATCAGC CTGGAAATGG ATAAACTGAA

1441 AATCGTGATT ACCAAACTGA CCATGCTGGC AGAACGTCAG CTGAATTATC TGCTGAATAG

1501 CAAAATTAAC GAACTGCTGC CTCCGTTTGT TAATCTGGGC ACCCTGGGTT TTAACTTTGG

1561 TATGCAGGGT GTTCAGTTTA CCGCAACCAG CACCACCGCA GAAAGCCAGA TGCTGAGCAA

1621 TCCGATGTAT GTTCATAGCA TTCCGAACAA TAATGATAAC CAGGATATTG TTAGCATGGG

1681 CACCAATAGC GCAGTTATTA CCAGCAAAGT TATCGAAAAT GCCTTTGAAG TTCTGGCCAT

1741 TGAAATGATT ACCATTGTTC AGGCGATTGA TTATCTGGGC CAGAAAGATA AAATCAGCAG

1801 CGTTAGCAAA AAATGGTATG ATGAAATCCG CAACATCATC CCGACCTTTA AAGAAGATCA

1861 GGTGATGTAT CCGTTCGTGC AGAAAGTAAA AGACCACCTG ATTAACAATT AATGACCATG

1921 GGCTAGCCTT CGTAAATCTG GCGAGTGGGG AACTGCCAGA CATCAAATAA AACAAAAGGC

1981 TCAGTCGGAA GACTGGGCCT TTTGTTTTAT CTGTTGTTTG TCGGTGAACA CTCTCCCAAA

2041 GACGAATTAC TTATCTGGCA GGAGTATCGC AAGGAAAAGT GCCACCTGAC GTCATTAATA

2101 GACGAATTAC TTATCTGGCA GGAGTATCGT CCGTAGTTTC AACTGTTCTG TGCATACGGC

2161 CCTGAAAGAC TATTGATTAC GAATATAAGG AAAACCACTA AATGAACGAT TTCTTAGTCG

2221 GCGTTATAGT AAGTCACTCT TTTTCAGCGG TATTTTAAAG ATGAGAAAGC GATGGTCAAG

2281 CGTGGTCTGC CTGAAGTCTC CTTCTAGAGC GCAGCTAACA CCACGTCGTC CCTATCTGCT

2341 GCCCTATGTT TAAAAAAATG TCGGAGAACG TGTTTATCAA ATGGTGCTGG GGTATGTAAC

2401 TTGTAGGGCC CAAGTTCACT TAAAAAGGAG ATCAACAATG AAAGCAATTT TCGTACTGAA

2461 ACATCTTAAT CATGCTATGG AGGTTTTCTA ATGGGTGACT GCGTTGCCCC GAAAGAGGAT

2521 CTGATCTTCC GCAGCAAACT GCCGGACATT TACATTCCAA AGCATCTGCC GCTGCATACG

2581 TATTGTTTTG AGAATATCAG CAAGGTTGGC GACAAGAGCT GTCTGATCAA CGGCGCAACC

2641 GGCGAAACGT TTACCTACAG CCAGGTCGAG CTGCTGTCCC GTAAAGTTGC CAGCGGCCTG

2701 AACAAGCTGG GCATTCAACA AGGTGATACC ATTATGCTGT TGCTGCCGAA TTCCCCGGAG

2761 TACTTTTTCG CTTTCCTGGG TGCGAGCTAT CGCGGTGCAA TCAGCACCAT GGCGAATCCA

2821 TTCTTTACCA GCGCAGAAGT GATCAAGCAA CTGAAAGCGA GCCAAGCGAA GCTGATTATC

2881 ACCCAGGCAT GCTATGTTGA CAAGGTCAAG GACTACGCAG CGGAGAAAAA CATCCAGATC

2941 ATTTGTATTG ACGATGCACC GCAGGATTGC CTGCACTTTA GCAAGCTGAT GGAAGCGGAT

3001 GAGAGCGAAA TGCCGGAAGT GGTCATTAAC AGCGATGATG TGGTGGCATT GCCGTACAGC

3061 TCTGGCACCA CCGGCCTGCC GAAAGGCGTT ATGCTGACCC ACAAGGGTCT GGTTACGAGC

3121 GTTGCACAAC AGGTGGATGG TGATAACCCG AACCTGTATA TGCACTCCGA GGATGTCATG

3181 ATCTGCATCC TGCCACTGTT CCATATCTAT AGCCTGAACG CTGTTCTGTG TTGTGGTCTG

3241 CGTGCGGGCG TCACCATTCT GATCATGCAA AAGTTCGACA TTGTGCCGTT TCTGGAGCTG

3301 ATTCAGAAGT ATAAGGTTAC GATTGGTCCG TTTGTCCCGC CGATCGTGCT GGCCATCGCG

3361 AAAAGCCCGG TCGTTGACAA GTACGACTTG TCTAGCGTGC GCACCGTCAT GAGCGGTGCA

3421 GCGCCGCTGG GTAAAGAGTT GGAGGACGCT GTCCGTGCGA AATTCCCGAA CGCGAAGCTG

3481 GGTCAAGGCT ATGGCATGAC CGAAGCCGGT CCGGTCCTGG CGATGTGTCT GGCGTTCGCC

3541 AAAGAGCCGT ATGAGATTAA GTCTGGCGCA TGCGGTACCG TTGTGCGTAA TGCCGAGATG

3601 AAAATCGTTG ACCCAGAAAC GAATGCGTCT CTGCCGCGTA ATCAGCGTGG TGAGATTTGC

3661 ATCCGTGGTG ATCAGATTAT GAAAGGTTAC CTGAATGACC CGGAAAGCAC CCGCACCACG

3721 ATCGACGAAG AGGGTTGGTT GCACACGGGT GACATTGGTT TCATCGACGA TGACGATGAA

3781 CTGTTCATTG TCGATCGTTT GAAAGAAATC ATTAAGTACA AAGGTTTTCA AGTTGCTCCG

3841 GCGGAGTTGG AAGCACTGCT GCTGACGCAC CCGACGATCA GCGATGCCGC GGTGGTTCCG

3901 ATGATTGACG AGAAAGCGGG TGAAGTGCCA GTGGCGTTTG TCGTGCGTAC CAATGGTTTT

3961 ACCACGACCG AAGAAGAAAT CAAACAATTT GTGAGCAAAC AGGTCGTGTT CTACAAACGT

4021 ATCTTCCGCG TCTTCTTCGT TGACGCTATT CCGAAATCCC CGAGCGGCAA GATTTTGCGT

4081 AAGGATCTGC GCGCTCGTAT TGCGAGCGGC GACCTGCCGA AGTAACCATG GGCTAGCATT

4141 GCCGTTCCGA CTGATTGGGG AACTGCCAGG CATCAAATAA AACGAAAGGC TCAGTCGAAA

4201 GACTGGGCCT TTCGTTTTAT CTGTTGTTTG TCGGTGAACG CTCTCCTGTC CTTATGTGGT

4261 GAGGGAATTA CCCTGTATTA GTGCTGTGGA AAAGTGCCAC CTGACGTCAT TAATTCCTTA

4321 TGTGGTGAGG GAATTACCCT GTATTAGTGC AACGGCGAGG AACGAAACTG GCGCGGACAT

4381 GATTTTGATT GATAATTGAA TGTTGCGGCT TCTACAATTT TCACAATTAT GTGAGTATTG

4441 TAAGCAATCA TGACATGATA AATTCTGATA ACGTGTCGGG TCATGTATTC AGCCAGTATG

4501 AGGAGCGCCT TCAGCCTTCT AGAGCGCAGC TAACACCACG TCGTCCCTAT CTGCTGCCCT

4561 ATGTTTAAAA AAATGTCGGA GAACGTGTTT ATGTTTTTTC TGTACAGGGT ATGTAACTTG

4621 TAGGGCCCAA GTTCACTTAA AAAGGAGATC AACAATGAAA GCAATTTTCG TACTGAAACA

4681 TCTTAATCAT GCTATGGAGG TTTTCTAATG GCGTCCTCCG TTGACATGAA GGCGATCAGA

4741 GATGCTCAAC GTGCAGAAGG TCCGGCGACC ATTCTTGCCA TCGGAACTGC AACTCCGGCG

4801 AATTGCGTCT ATCAAGCGGA TTATCCCGAT TACTATTTTC GGATCACCAA GAGTGAACAC

4861 ATGGTGGATC TCAAAGAGAA ATTCAAGCGC ATGTGTGACA AGTCGATGAT AAGGAAACGT

4921 TACATGCACA TCACAGAGGA GTATCTTAAA CAAAACCCTA ACATGTGCGC GTACATGGCG

4981 CCGTCGCTCG ACGTCCGGCA AGACCTGGTC GTCGTCGAAG TCCCAAAGCT CGGCAAGGAA

5041 GCCGCCATGA AAGCCATCAA AGAATGGGGC CACCCCAAAT CCAAGATCAC CCACCTCATC

5101 TTCTGCACCA CCTCCGGCGT CGACATGCCC GGCGCCGACT ACCAGCTCAC CAAACTCCTC

5161 GGCCTCCGGC CATCCGTCAA ACGCTTCATG ATGTACCAAC AAGGCTGCTT CGCCGGCGGC

5221 ACGGTTCTCC GGCTAGCCAA AGATCTCGCG GAGAACAATA AAGGCGCTAG GGTTCTTGTG

5281 GTGTGCTCCG AGATCACGGC GGTGACTTTC CGAGGACCTA ATGACACCCA CCTTGATTCC

5341 CTAGTCGGAC AGGCCTTGTT CGGCGACGGG GCTGCGGCGG TGATCGTGGG TTCCGATCCC

5401 GACTTGACGA CGGAGCGGCC GTTGTTTGAA ATGGTTTCCG CCGCTCAGAC GATCTTGCCG

5461 GACTCCGAGG GAGCCATTGA TGGACACTTG AGGGAAGTAG GGTTGACGTT TCATTTACTC

5521 AAAGACGTGC CTGGGTTGAT ATCGAAGAAC ATAGAGAAAG CTTTAACGAC GGCGTTTTCT

5581 CCGTTGGGTA TCAACGACTG GAACTCGATA TTCTGGATAG CACATCCCGG AGGTCCGGCG

5641 ATACTGGACC AGGTGGAGCT CAAGCTAGGG TTGAAGGAGG AGAAGCTTAG AGCTACTAGA

5701 CATGTTTTAA GCGAGTACGG TAACATGTCA AGTGCTTGTG TGTTGTTTAT TATCGACGAA

5761 ATGAGAAAGA AGTCGTCGGA GAACGGCGCC GGCACCACCG GAGAAGGTTT GGAGTGGGGT

5821 GTTCTGTTTG GGTTTGGGCC TGGGTTGACG GTGGAGACGG TGGTTCTTCA CAGTGTCCCA

5881 ACCACCGTGA CGGTTCCCGT CTAACCATGG GCTAGCGGCC AGCAAAGGGA CAAAGCTTCA

5941 CCATGCGTAA AGCAATCAGA TACCCAGCCC GCCTAATAAG CGGGCTTTTT TTTGAACAAA

6001 ATTAGAGAAT AACAATGCAA ACATGCAATT ACTACCATTA AATAATTAGT CAGGCGTCGA

6061 GTACCATATG ACGAAAAGTG CCACCTGACG TCATTAATTG CAATTACTAC CATTAAATAA

6121 TTAGTCAGGC GTCGAGTACC ATAAGATTGC CGCAGCGCTT TATTGAAGAA CTGCTCGCTA

6181 AGATACTTAC TAAATGACAA AGTTGCAGAG AGAGTTTAAC CGTGAGTTAA TTCTCAGAGG

6241 GTGCGCCCGC TTTACATCGC TTCAGTGCTT GTACCCATCT GATGCACGCC ATCGGAACCC

6301 TTCTAGAGCG CAGCTAACAC CACGTCGTCC CTATCTGCTG CCCTATGTTT AAAAAAATGT

6361 CGGAGAACGT GTTTATCAAA TGGTGCTGGG GTATGTAACT TGTAGGGCCC AAGTTCACTT

6421 AAAAAGGAGA TCAACAATGA AAGCAATTTT CGTACTGAAA CATCTTAATC ATGCTATGGA

6481 GGTTTTCTAA TGGCTGCATC AATCACCGCA ATCACTGTGG AGAACCTTGA ATACCCAGCG

6541 GTGGTTACCT CTCCGGTCAC CGGCAAATCA TATTTCCTCG GTGGCGCTGG GGAGAGAGGA

6601 TTGACCATTG AAGGAAACTT CATCAAGTTC ACTGCCATAG GTGTTTATTT GGAAGATATA

6661 GCAGTGGCTT CACTAGCTGC CAAATGGAAG GGTAAATCAT CTGAAGAGTT ACTTGAAACC

6721 CTTGACTTTT ACAGAGACAT CATCTCAGGT CCCTTTGAAA AGTTAATTAG AGGGTCAAAG

6781 ATTAGGGAAT TGAGTGGTCC TGAGTACTCA AGGAAGGTTA TGGAGAACTG TGTGGCACAC

6841 TTGAAATCAG TTGGAACTTA TGGAGATGCA GAAGCTGAAG CTATGCAAAA ATTTGCTGAA

6901 GCTTTCAAGC CTGTTAATTT TCCACCTGGT GCCTCTGTTT TCTACAGGCA ATCACCTAAT

6961 GGAATATTAG GGCTTAGTTT CTCTCCGGAT ACAAGTATAC CAGAAAAGGA GGCTGCACTC

7021 ATAGAGAACA AGGCAGTTTC ATCAGCAGTG TTGGAGACTA TGATCGGCGA GCACGCTGTT

7081 TCCCCTGATC TTAAGCGCTG TTTAGCTGCA AGATTACCTG CGTTGTTGAA CGAGGGTGCT

7141 TTCAAGATTG GAAACTGACC ATGGGCTAGC AATGCCAAAC GGCCTTAACC ACAACCCTCA

7201 AGAGAAAATG TAATCACACT GGCTCACCTT CGGGTGGGCC TTTCTGCGTT TATAAGGAGA

7261 CACTTTATGT TTAAGAAGTG CATGATATTA TCGCACGGCT CCAAGTCCTA GGATGCTAGC

7321 TATGTGGGCT TACATGGCGA TAGCTAGACT GGGCGGTTTT ATGGACAGCA AGCGAACCGG

7381 AATTGCCAGC TGGGGCGCCC TCTGGTAAGG TTGGGAAGCC CTGCAAAGTA AACTGGATGG

7441 CTTTCTTGCC GCCAAGGATC TGATGGCGCA GGGGATCAAG ATCTGATCAA GAGACAGGAT

7501 GAGGATCGTT TCGCATGATT GAACAAGATG GATTGCACGC AGGTTCTCCG GCCGCTTGGG

7561 TGGAGAGGCT ATTCGGCTAT GACTGGGCAC AACAGACAAT CGGCTGCTCT GATGCCGCCG

7621 TGTTCCGGCT GTCAGCGCAG GGGCGCCCGG TTCTTTTTGT CAAGACCGAC CTGTCCGGTG

7681 CCCTGAATGA ACTGCAGGAC GAGGCAGCGC GGCTATCGTG GCTGGCCACG ACGGGCGTTC

7741 CTTGCGCAGC TGTGCTCGAC GTTGTCACTG AAGCGGGAAG GGACTGGCTG CTATTGGGCG

7801 AAGTGCCGGG GCAGGATCTC CTGTCATCTC ACCTTGCTCC TGCCGAGAAA GTATCCATCA

7861 TGGCTGATGC AATGCGGCGG CTGCATACGC TTGATCCGGC TACCTGCCCA TTCGACCACC

7921 AAGCGAAACA TCGCATCGAG CGAGCACGTA CTCGGATGGA AGCCGGTCTT GTCGATCAGG

7981 ATGATCTGGA CGAAGAGCAT CAGGGGCTCG CGCCAGCCGA ACTGTTCGCC AGGCTCAAGG

8041 CGCGCATGCC CGACGGCGAG GATCTCGTCG TGACCCATGG CGATGCCTGC TTGCCGAATA

8101 TCATGGTGGA AAATGGCCGC TTTTCTGGAT TCATCGACTG TGGCCGGCTG GGTGTGGCGG

8161 ACCGCTATCA GGACATAGCG TTGGCTACCC GTGATATTGC TGAAGAGCTT GGCGGCGAAT

8221 GGGCTGACCG CTTCCTCGTG CTTTACGGTA TCGCCGCTCC CGATTCGCAG CGCATCGCCT

8281 TCTATCGCCT TCTTGACGAG TTCTTCTGAG CGGGACTCTG GGGTTCGAAA TGACCGACCA

8341 AGCGACGCCC AACCTGCCAT CACGAGATTT CGATTCCACC GCCGCCTTCC CCCCATGAAC

8401 AGAAATCCCC CTTACACGGA GGCATCAGTG ACCAAACAGG AAAAAACCGC CCTTAACATG

8461 GCCCGCTTTA TCAGAAGCCA GACATTAACG CTTCTGGAGA AACTCAACGA GCTGGACGCG

8521 GATGAACAGG CAGACATCTG TGAATCGCTT CACGACCACG CTGATGAGCT TTACCGCAGC

8581 TGCCTCGCGC GTTTCGGTGA TGACGGTGAA AACCTCTGAC ACATGCAGCT CCCGGAGACG

8641 GTCACAGCTT GTCTGTAAGC GGATGCCGGG AGCAGACAAG CCCGTCAGGG CGCGTCAGCG

8701 GGTGTTGGCG GGTGTCGGGG CGCAGCCATG ACCCAGTCAC GTAGCGATAG CGGAGTGTAT

8761 ACTGGCTTAA CTATGCGGCA TCAGAGCAGA TTGTACTGAG AGTGCACCAT ATGCGGTGTG

8821 AAATACCGCA CAGATGCGTA AGGAGAAAAT ACCGCATCAG GCGCTCTTCC GCTTCCTCGC

8881 TCACTGACTC GCTGCGCTCG GTCGTTCGGC TGCGGCGAGC GGTATCAGCT CACTCAAAGG

8941 CGGTAATACG GTTATCCACA GAATCAGGGG ATAACGCAGG AAAGAACATG TGAGCAAAAG

9001 GCCAGCAAAA GGCCAGGAAC CGTAAAAAGG CCGCGTTGCT GGCGTTTTTC CATAGGCTCC

9061 GCCCCCCTGA CGAGCATCAC AAAAATCGAC GCTCAAGTCA GAGGTGGCGA AACCCGACAG

9121 GACTATAAAG ATACCAGGCG TTTCCCCCTG GAAGCTCCCT CGTGCGCTCT CCTGTTCCGA

9181 CCCTGCCGCT TACCGGATAC CTGTCCGCCT TTCTCCCTTC GGGAAGCGTG GCGCTTTCTC

9241 ATAGCTCACG CTGTAGGTAT CTCAGTTCGG TGTAGGTCGT TCGCTCCAAG CTGGGCTGTG

9301 TGCACGAACC CCCCGTTCAG CCCGACCGCT GCGCCTTATC CGGTAACTAT CGTCTTGAGT

9361 CCAACCCGGT AAGACACGAC TTATCGCCAC TGGCAGCAGC CACTGGTAAC AGGATTAGCA

9421 GAGCGAGGTA TGTAGGCGGT GCTACAGAGT TCTTGAAGTG GTGGCCTAAC TACGGCTACA

9481 CTAGAAGGAC AGTATTTGGT ATCTGCGCTC TGCTGAAGCC AGTTACCTTC GGAAAAAGAG

9541 TTGGTAGCTC TTGATCCGGC AAACAAACCA CCGCTGGTAG CGGTGGTTTT TTTGTTTGCA

9601 AGCAGCAGAT TACGCGCAGA AAAAAAGGAT CTCAAGAAGA TCCTTTGATC TTTTCTACGG

9661 GGTCTGACGC TCAGTGGAAC GAAAACTCAC GTTAAGGGAT TTTGGTCATG AGATTATCAA

9721 AAAGGATCTT CACCTAGATC CTTTTAAATT AAAAATGAAG TTTTAAATCA ATCTAAAGTA

9781 TATATGAGTA AACTTGGTCT GACAGAGCTG GCACGACAGG TTTCCCGACT GGAAATAGAC

9841 GTCGCCTCAG CTTGCATCGA AAAGTGCCAC CTGACGTCAT TAAT

//

**Additional file 1: Figure S12:** Annotated genbank file of the optimized naringenin biosynthesis pathway (pTop10.4, Figure 6). The expression vector originates from Coussement *et al*. (2017) (34) (Table 1). The promoters driving the pathway are created in Bervoets *et al.* (2018) (8). More information about the enzymes and source of CDSs can be found in Table 2. The used transcription terminators are from the BIOFAB collection (72).

**BIBLIOGRAPHY**

1. Wargacki AJ, Leonard E, Win MN, Regitsky DD, Santos CNS, Kim PB, et al. An engineered microbial platform for direct biofuel production from brown macroalgae. Science. 2012 Jan;335(6066):308–13.

2. Cheon S, Kim HM, Gustavsson M, Lee SY. Recent trends in metabolic engineering of microorganisms for the production of advanced biofuels. Curr Opin Chem Biol. 2016 Dec;35:10–21.

3. Pandey RP, Parajuli P, Koffas MAG, Sohng JK. Microbial production of natural and non-natural flavonoids: Pathway engineering, directed evolution and systems/synthetic biology. Biotechnol Adv. 2016 Sep;34(5):634–62.

4. Paddon CJ, Keasling JD. Semi-synthetic artemisinin: A model for the use of synthetic biology in pharmaceutical development. Nat Rev Microbiol. 2014 May;12(5):355–67.

5. Trantas EA, Koffas MAG, Xu P, Ververidis F. When plants produce not enough or at all: Metabolic engineering of flavonoids in microbial hosts. Front Plant Sci. 2015 Jan;6:7.

6. Keasling JD. Manufacturing molecules through metabolic engineering. Science. 2010 Dec;330(6009):1355–8.

7. Woolston BM, Edgar S, Stephanopoulos G. Metabolic engineering: past and future. Annu Rev Chem Biomol Eng. 2013 Jan 7;4:259–88.

8. Bervoets I, Van Brempt M, Van Nerom K, Van Hove B, Maertens J, De Mey M, et al. A sigma factor toolbox for orthogonal gene expression in *Escherichia coli*. Nucleic Acids Res. 2018 Feb;46(4):2133–44.

9. Cheng S, Liu X, Jiang G, Wu J, Zhang JL, Lei D, et al. Orthogonal engineering of biosynthetic pathway for efficient production of limonene in *Saccharomyces cerevisiae*. ACS Synth Biol. 2019 May 17;8(5):968–75.

10. Pandit AV, Srinivasan S, Mahadevan R. Redesigning metabolism based on orthogonality principles. Nat Commun 2017 81. 2017 May 30;8(1):1–11.

11. Snoek T, Romero-Suarez D, Zhang J, Ambri F, Skjoedt ML, Sudarsan S, et al. An orthogonal and pH-tunable sensor-selector for muconic acid biosynthesis in yeast. ACS Synth Biol. 2018 Apr 20;7(4):995–1003.

12. Haushalter RW, Groff D, Deutsch S, The L, Chavkin TA, Brunner SF, et al. Development of an orthogonal fatty acid biosynthesis system in *E. coli* for oleochemical production. Metab Eng. 2015 Jul 1;30:1–6.

13. Falcone Ferreyra ML, Rius SP, Casati P. Flavonoids: biosynthesis, biological functions, and biotechnological applications. Front Plant Sci. 2012;3:222.

14. Panche AN, Diwan AD, Chandra SR. Flavonoids: an overview. J Nutr Sci. 2016 Dec 29;5:e47.

15. Bolwell GP, Bozak K, Zimmerlin A. Plant cytochrome P450. Phytochemistry. 1994 Dec;37(6):1491–506.

16. Delmulle T, De Maeseneire SL, De Mey M. Challenges in the microbial production of flavonoids. Phytochem Rev. 2018 Apr;17(2):229–47.

17. Wang Y, Chen S, Yu O. Metabolic engineering of flavonoids in plants and microorganisms. Appl Microbiol Biotechnol. 2011 Aug 6;91(4):949–56.

18. Forkmann G, Martens S. Metabolic engineering and applications of flavonoids. Curr Opin Biotechnol. 2001;12:155–60.

19. Santos CNS, Koffas M, Stephanopoulos G. Optimization of a heterologous pathway for the production of flavonoids from glucose. Metab Eng. 2011 Jul;13(4):392–400.

20. Leonard E, Yan Y, Fowler ZL, Li Z, Lim CG, Lim KH, et al. Strain improvement of recombinant *Escherichia coli* for efficient production of plant flavonoids. Mol Pharm. 2008 Jan;5(2):257–65.

21. Kaneko M, Hwang E Il, Ohnishi Y, Horinouchi S. Heterologous production of flavanones in *Escherichia coli*: Potential for combinatorial biosynthesis of flavonoids in bacteria. J Ind Microbiol Biotechnol. 2003;30(8):456–61.

22. Wu J, Zhou T, Du G, Zhou J, Chen J. Modular optimization of heterologous pathways for *de novo* synthesis of (2S)-naringenin in *Escherichia coli*. PLoS One. 2014 Jul 2;9(7):e101492.

23. Wu J, Du G, Zhou J, Chen J. Systems metabolic engineering of microorganisms to achieve large-scale production of flavonoid scaffolds. J Biotechnol. 2014 Aug 23;188:72–80.

24. Wu J, Du G, Chen J, Zhou J. Enhancing flavonoid production by systematically tuning the central metabolic pathways based on a CRISPR interference system in *Escherichia coli*. Sci Rep. 2015 Jan;5:13477.

25. Zhou S, Lyu Y, Li H, Koffas MAG, Zhou J. Fine-tuning the (2S)-naringenin synthetic pathway using an iterative high-throughput balancing strategy. Biotechnol Bioeng. 2019 Jun 1;116(6):1392–404.

26. Jones JA, Toparlak TD, Koffas MAG. Metabolic pathway balancing and its role in the production of biofuels and chemicals. Curr Opin Biotechnol. 2015;33:52–9.

27. Ceroni F, Algar R, Stan G-B, Ellis T. Quantifying cellular capacity identifies gene expression designs with reduced burden. Nat Methods. 2015 May;12(5):415–8.

28. Wu G, Yan Q, Jones JA, Tang YJ, Fong SS, Koffas MAG. Metabolic burden: Cornerstones in synthetic biology and metabolic engineering applications. Trends Biotechnol. 2016 Aug;34(8):652–64.

29. Biggs BW, De Paepe B, Santos CNS, De Mey M, Kumaran Ajikumar P. Multivariate modular metabolic engineering for pathway and strain optimization. Curr Opin Biotechnol. 2014 Oct;29(1):156–62.

30. Zhou Y, Li G, Dong J, Xing X hui, Dai J, Zhang C. MiYA, an efficient machine-learning workflow in conjunction with the YeastFab assembly strategy for combinatorial optimization of heterologous metabolic pathways in *Saccharomyces cerevisiae*. Metab Eng. 2018 May;47:294–302.

31. Jervis AJ, Carbonell P, Vinaixa M, Dunstan MS, Hollywood KA, Robinson CJ, et al. Machine learning of designed translational control allows predictive pathway optimization in *Escherichia coli*. ACS Synth Biol. 2019 Jan 18;8(1):127–36.

32. Liu R, Bassalo MC, Zeitoun RI, Gill RT. Genome scale engineering techniques for metabolic engineering. Metab Eng. 2015 Oct 9;32:143–54.

33. De Paepe B, Peters G, Coussement P, Maertens J, De Mey M. Tailor-made transcriptional biosensors for optimizing microbial cell factories. J Ind Microbiol Biotechnol. 2017 May 11;44(4–5):623–45.

34. Coussement P, Bauwens D, Maertens J, De Mey M. Direct combinatorial pathway optimization. ACS Synth Biol. 2017 Feb;6(2):224–32.

35. Zou R, Zhou K, Stephanopoulos G, Too HP. Combinatorial engineering of 1-deoxy-D-xylulose 5-phosphate pathway using cross-lapping *in vitro* assembly (CLIVA) method. PLoS One. 2013 Nov;8(11):e79557.

36. De Paepe B, Maertens J, Vanholme B, De Mey M. Modularization and response curve engineering of a Naringenin-responsive transcriptional biosensor. ACS Synth Biol. 2018 May;7(5):1303–14.

37. Farasat I, Kushwaha M, Collens J, Easterbrook M, Guido M, Salis HM. Efficient search, mapping, and optimization of multi-protein genetic systems in diverse bacteria. Mol Syst Biol. 2014;10:731.

38. Xu P, Rizzoni EA, Sul S-Y, Stephanopoulos G. Improving metabolic pathway efficiency by statistical model-based multivariate regulatory metabolic engineering. ACS Synth Biol. 2017 Jan;6(1):148–58.

39. Engler C, Kandzia R, Marillonnet S. A one pot, one step, precision cloning method with high throughput capability. PLoS One. 2008 Jan;3(11):e3647.

40. Lee ME, Aswani A, Han AS, Tomlin CJ, Dueber JE. Expression-level optimization of a multi-enzyme pathway in the absence of a high-throughput assay. Nucleic Acids Res. 2013;41(22):10668–78.

41. Zhou H, Vonk B, Roubos JA, Bovenberg RAL, Voigt CA. Algorithmic co-optimization of genetic constructs and growth conditions: Application to 6-ACA, a potential nylon-6 precursor. Nucleic Acids Res. 2015 Oct;43(21):gkv1071.

42. Wold S, Sjöström M, Eriksson L. PLS-regression: A basic tool of chemometrics. Chemom Intell Lab Syst. 2001 Oct;58(2):109–30.

43. Alonso-Gutierrez J, Kim E-M, Batth TS, Cho N, Hu Q, Chan LJG, et al. Principal component analysis of proteomics (PCAP) as a tool to direct metabolic engineering. Metab Eng. 2015 Mar;28:123–33.

44. Jonsson J, Norberg T, Carlsson L, Gustafsson C, Wold S. Quantitative sequence-activity models (QSAM)--tools for sequence design. Nucleic Acids Res. 1993 Feb;21(3):733–9.

45. Mutalik VK, Qi L, Guimaraes JC, Lucks JB, Arkin AP. Rationally designed families of orthogonal RNA regulators of translation. Nat Chem Biol. 2012;8(5):447–54.

46. De Mey M, Maertens J, Lequeux GJ, Soetaert WK, Vandamme EJ. Construction and model-based analysis of a promoter library for *E. coli*: An indispensable tool for metabolic engineering. BMC Biotechnol. 2007;7:34.

47. Grubbs FE. Sample criteria for testing outlying observations. Ann Math Stat. 1950 Mar;21(1):27–58.

48. De Mey M, Maertens J, Lequeux GJ, Soetaert WK, Vandamme EJ. Construction and model-based analysis of a promoter library for *E. coli*: An indispensable tool for metabolic engineering. BMC Biotechnol. 2007;7(1):34.

49. Ahn JO, Lee HW, Saha R, Park MS, Jung J-K, Lee D-Y. Exploring the effects of carbon sources on the metabolic capacity for shikimic acid production in *Escherichia coli* using *in silico* metabolic predictions. J Microbiol Biotechnol. 2008 Nov;18(11):1773–84.

50. Yang D, Kim WJ, Yoo SM, Choi JH, Ha SH, Lee MH, et al. Repurposing type III polyketide synthase as a malonyl-CoA biosensor for metabolic engineering in bacteria. Proc Natl Acad Sci U S A. 2018 Oct;115(40):9835–44.

51. Lyu X, Ng KR, Lee JL, Mark R, Chen WN. Enhancement of naringenin biosynthesis from tyrosine by metabolic engineering of *Saccharomyces cerevisiae*. J Agric Food Chem. 2017;65(31):6638–46.

52. Wainwright M, Canham LT, Al-Wajeeh K, Reeves CL. Morphological changes (including filamentation) in *Escherichia coli* grown under starvation conditions on silicon wafers and other surfaces. Lett Appl Microbiol. 1999 Oct;29(4):224–7.

53. Kosuri S, Goodman DB, Cambray G, Mutalik VK, Gao Y, Arkin AP, et al. Composability of regulatory sequences controlling transcription and translation in *Escherichia coli*. Proc Natl Acad Sci U S A. 2013 Aug;110(34):14024–9.

54. Hausser J, Mayo A, Keren L, Alon U. Central dogma rates and the trade-off between precision and economy in gene expression. Nat Commun. 2019 Dec;10(1):68.

55. Zha W, Rubin-Pitel SB, Shao Z, Zhao H. Improving cellular malonyl-CoA level in *Escherichia coli* via metabolic engineering. Metab Eng. 2009 May;11(3):192–8.

56. Fowler ZL, Gikandi WW, Koffas MAG. Increased malonyl Coenzyme A biosynthesis by tuning the *Escherichia coli* metabolic network and its application to flavanone production. Appl Environ Microbiol. 2009 Sep;75(18):5831–9.

57. Xu P, Ranganathan S, Fowler ZL, Maranas CD, Koffas MAG. Genome-scale metabolic network modeling results in minimal interventions that cooperatively force carbon flux towards malonyl-CoA. Metab Eng. 2011 Sep;13(5):578–87.

58. Lou H, Hu L, Lu H, Wei T, Chen Q. Metabolic engineering of microbial cell factories for biosynthesis of flavonoids: A Review. Mol 2021, Vol 26, Page 4522. 2021 Jul 27;26(15):4522.

59. Dunstan MS, Robinson CJ, Jervis AJ, Yan C, Carbonell P, Hollywood KA, et al. Engineering *Escherichia coli* towards de novo production of gatekeeper (2S)-flavanones: naringenin, pinocembrin, eriodictyol and homoeriodictyol. Synth Biol. 2020 Jan 1;5(1).

60. Zhou S, Hao T, Zhou J. Fermentation and metabolic pathway optimization to de novo synthesize (2S)-Naringenin in *Escherichia coli*. J Microbiol Biotechnol. 2020 Oct 28;30(10):1574–82.

61. Zhou S, Yuan SF, Nair PH, Alper HS, Deng Y, Zhou J. Development of a growth coupled and multi-layered dynamic regulation network balancing malonyl-CoA node to enhance (2S)-naringenin biosynthesis in *Escherichia coli*. Metab Eng. 2021 Sep 1;67:41–52.

62. Sambrook, J., E. F. Fritsch and TM. Molecular cloning: a laboratory manual, 2nd ed. Cold Spring Harbor Laboratory, Cold Spring Harbor, N.Y.; 1989.

63. Zhu S, Wu J, Du G, Zhou J, Chen J. Efficient synthesis of eriodictyol from L-tyrosine in *Escherichia coli*. Appl Environ Microbiol. 2014 May;80(10):3072–80.

64. Vannelli T, Xue Z, Breinig S, Qi WW, Sariaslani FS. Functional expression in *Escherichia coli* of the tyrosine-inducible tyrosine ammonia-lyase enzyme from yeast *Trichosporon cutaneum* for production of p-hydroxycinnamic acid. Enzyme Microb Technol. 2007 Sep;41(4):413–22.

65. Rodrigues JL, Araújo RG, Prather KLJ, Kluskens LD, Rodrigues LR. Heterologous production of caffeic acid from tyrosine in *Escherichia coli*. Enzyme Microb Technol. 2015 Apr;71:36–44.

66. Jendresen CB, Stahlhut SG, Li M, Gaspar P, Siedler S, Förster J, et al. Highly active and specific tyrosine ammonia-lyases from diverse origins enable enhanced production of aromatic compounds in bacteria and *Saccharomyces cerevisiae*. Appl Environ Microbiol. 2015 Jul;81(13):4458–76.

67. Leonard E, Chemler J, Lim KH, Koffas MAG. Expression of a soluble flavone synthase allows the biosynthesis of phytoestrogen derivatives in *Escherichia coli*. Appl Microbiol Biotechnol. 2006 Mar;70(1):85–91.

68. Leonard E, Yan Y, Koffas MAG. Functional expression of a P450 flavonoid hydroxylase for the biosynthesis of plant-specific hydroxylated flavonols in *Escherichia coli*. Metab Eng. 2006;8(2):172–81.

69. Helariutta Y, Elomaa P, Kotilainen M, Griesbach RJ, Schröder J, Teeri TH. Chalcone synthase-like genes active during corolla development are differentially expressed and encode enzymes with different catalytic properties in *Gerbera hybrida* (Asteraceae). Plant Mol Biol. 1995 Apr;28(1):47–60.

70. Mevik B-H, Wehrens R. The pls package: Principal component and partial least squares regression in R. J Stat Softw. 2007 Jan;18(2):1–23.

71. Revelle W. psych: Procedures for psychological, psychometric, and personality research, Northwestern University, Evanston, Illinois, USA. Evanston, Illinois; 2018.

72. Mutalik VK, Guimaraes JC, Cambray G, Lam C, Christoffersen MJ, Mai Q-A, et al. Precise and reliable gene expression via standard transcription and translation initiation elements. Nat Methods. 2013;10(4):354–60.
